# Insights into vitamin C in musculoskeletal physiology and disorders: mechanisms and translational perspectives

**DOI:** 10.3389/fimmu.2026.1899148

**Published:** 2026-08-17

**Authors:** Xu Liu, Bo Chen, Qingnan Feng, Shitong Wang, Zhengxu Fu, Jingdong Wu, Xiaohong Chen, Xin Zhang

**Affiliations:** 1 The First Clinical College, Liaoning University of Traditional Chinese Medicine, Shenyang, China; 2 Department of Exercise Physiology, Beijing Sport University, Beijing, China; 3 Guangdong Provincial Key Laboratory of Physical Activity and Health Promotion, Guangzhou Sport University, Guangzhou, China; 4 The First Clinical College, Shenyang Medical University, Shenyang, China; 5 Department of Gerontology, Jin Qiu Hospital of Liaoning Province, Geriatric Hospital of Liaoning Province, Shenyang, China; 6 Department of Rehabilitation Medicine, Shenyang First People’s Hospital, Shenyang, China

**Keywords:** vitamin C, musculoskeletal system, osteoporosis, osteoarthritis, rheumatoid arthritis, sarcopenia, exercise

## Abstract

Vitamin C (VC) is an essential micronutrient involved in diverse biological processes, including redox homeostasis, energy metabolism, collagen synthesis, epigenetic regulation, and immune modulation. Increasing evidence indicates that VC plays important roles in the musculoskeletal system, which is essential for structural support, locomotion, and systemic metabolic homeostasis. This review provides an integrated overview of current evidence regarding the roles of VC in musculoskeletal physiology and disorders, with a focus on muscle development and regeneration, bone formation and remodeling, and tendon–bone repair. We further discuss the potential involvement of VC in major musculoskeletal disorders (including osteoporosis, osteoarthritis, rheumatoid arthritis, and sarcopenia) and summarize the interactions between VC supplementation and exercise in musculoskeletal health. By integrating findings from cellular, animal, and human studies, this review highlights that current evidence remains strongest at the mechanistic and preclinical levels, whereas direct clinical evidence and translational validation remain limited. These insights may help guide future experimental studies, more rigorous clinical trials, and the development of VC-based strategies for musculoskeletal health.

## 1 Introduction

The musculoskeletal system, the largest organ system in the human body, comprises muscles, bones, cartilage, joints, tendons, and associated connective tissues. It not only provides structural support and enables movement but also plays a critical role in systemic metabolic regulation (1). Pathological conditions affecting this system, including osteoporosis (OP), osteoarthritis (OA), rheumatoid arthritis (RA), and sarcopenia, represent major musculoskeletal disorders (2). Although associated with relatively low mortality, the pain, mobility limitations, and functional impairments caused by these disorders severely diminish quality of life, establishing them as the leading cause of disability worldwide (3–5). Over the past three decades, the global burden of musculoskeletal disorders and the associated healthcare costs have risen steadily because of population aging (6). By 2035, the number of individuals affected by musculoskeletal disorders will reach 2.161 billion, accompanied by further increases in disability and mortality (7). Therefore, identifying effective strategies for the prevention and management of musculoskeletal disorders is of considerable importance.

Nutrition plays an essential role in maintaining musculoskeletal health (8). Vitamin C (VC), also known as ascorbic acid, is a water-soluble essential micronutrient with diverse physiological functions (9). As a potent reducing agent and an enzymatic cofactor, VC participates in antioxidant defense, carnitine and collagen biosynthesis, and epigenetic regulation. These biochemical properties provide a mechanistic basis for its involvement in the development, homeostasis, and repair of musculoskeletal tissues (10–12). Although growing evidence links VC to musculoskeletal physiology and disorders, the precise molecular mechanisms underlying its actions remain incompletely understood, and the translational relevance of current findings has not been fully established. Moreover, an updated integrative review that connects mechanistic insights with disease-specific evidence and exercise-related implications is still needed. Therefore, this review summarizes the roles of VC in musculoskeletal physiology; evaluates its potential involvement in OP, OA, RA, and sarcopenia; and discusses the interaction between VC supplementation and exercise in musculoskeletal health. By integrating evidence from basic, preclinical, and clinical studies, this review aims to provide a balanced perspective on the biological significance and translational relevance of VC in musculoskeletal health and disorders.

## 2 Literature search strategy

A structured literature search was conducted in PubMed and Web of Science from database inception to July 6, 2026. Search terms included VC and related forms or transporters, such as “VC,” “ascorbic acid,” “ascorbate,” “dehydroascorbic acid,” “SVCT,” “SVCT1,” and “SVCT2,” combined with terms related to the musculoskeletal system, including “skeletal muscle,” “bone,” “cartilage,” “tendon,” “fracture healing,” “osteoporosis,” “osteoarthritis,” “rheumatoid arthritis,” “sarcopenia,” and “exercise.” Additional records were identified by manually screening the reference lists of relevant original articles, reviews, meta-analyses, and clinical studies.

Studies were eligible for inclusion if they met at least one of the following criteria: (i) investigated the biological roles or molecular mechanisms of VC in musculoskeletal tissues or cells, including skeletal muscle cells, bone cells, chondrocytes, tenocytes, or mesenchymal stem cells; (ii) evaluated VC status, dietary intake, or supplementation in relation to musculoskeletal outcomes in observational or interventional human studies; (iii) assessed the effects of VC in animal models of musculoskeletal disorders, repair, or regeneration; or (iv) examined interactions between VC supplementation and exercise-induced musculoskeletal adaptation. Studies were excluded if they focused exclusively on non-musculoskeletal tissues without clear relevance to musculoskeletal physiology or disease, reported VC metabolism without musculoskeletal outcomes or mechanisms, lacked full-text availability, or were conference abstracts, editorials, letters, or non-peer-reviewed reports.

## 3 Overview of VC

VC is a ubiquitous, small, water-soluble carbohydrate. Since its first chemical synthesis in 1933, its role in human health has remained a major focus of scientific investigation (13). Unlike most mammals, humans cannot synthesize VC endogenously because of functional mutations and deletions in the gene encoding L-gulonolactone oxidase (GULO), which catalyzes the final step of VC biosynthesis (14). Therefore, VC must be obtained from dietary sources, particularly fruits and vegetables such as guava, blackcurrant, kiwifruit, broccoli, kale, and peppers (15). VC exerts diverse biological functions through enzymatic and non-enzymatic mechanisms. As a key cofactor for monooxygenases and dioxygenases, VC participates in collagen and carnitine synthesis, hormone production, tyrosine metabolism, and transcriptional regulation. It also regulates gene expression by promoting DNA and histone demethylation and by supporting the hydroxylation of transcription factors, transfer RNAs, and ribosomal proteins (16). In its non-enzymatic role, VC is renowned for its potent antioxidant properties, protecting cellular macromolecules such as DNA, proteins, and lipids from oxidative damage, while also exerting anti-inflammatory effects by reducing circulating pro-inflammatory cytokines (17, 18). VC also interacts with other antioxidants, particularly vitamin E, by regenerating the reduced form of vitamin E at the lipid–water interface, thereby forming a synergistic antioxidant network (19). VC exists predominantly as ascorbate, the reduced form of VC at physiological pH, and can be oxidized via the relatively stable ascorbyl radical to dehydroascorbic acid (DHA), its two-electron oxidation product. DHA can be taken up by many cell types and recycled back to ascorbate through glutathione-dependent or NADPH-dependent reduction, thereby limiting VC loss. Notably, the known biological functions of VC are linked to its reduced form (20).

The pharmacokinetics of VC are tightly regulated by intestinal absorption, tissue distribution, renal reabsorption, and excretion (21). Systemic VC homeostasis is maintained by passive diffusion, facilitated diffusion, and active transport, among which sodium-dependent VC transporters (SVCTs) play a central role (22, 23). SVCT1 mediates active intestinal absorption of ascorbate and renal reabsorption when body VC reserves and plasma VC concentrations are low, thereby minimizing urinary loss (24). DHA can also be absorbed in small amounts through glucose transporter-mediated facilitated diffusion (21). Tissue distribution is regulated mainly by SVCT2, which concentrates VC from plasma levels of approximately 50–80 μmol/L to tissue concentrations of 0.5–10 mmol/L (25, 26). Under physiological conditions, passive diffusion contributes little to VC homeostasis (22). Circulating VC status is commonly classified as deficient at <11 μmol/L, hypovitaminosis C at 11–23 μmol/L, inadequate at 23–49 μmol/L, adequate at 50–69 μmol/L, and saturating at ≥70 μmol/L (27). VC status is influenced by smoking, alcohol use, aging, obesity, metabolic disorders, inflammation, infection, trauma, and other conditions associated with oxidative stress. Chronic VC deficiency compromises connective tissue integrity and wound healing and may cause anemia, fatigue, and, in severe cases, scurvy. Although generally safe, excessive VC intake can cause gastrointestinal discomfort and increase urinary oxalate levels, potentially predisposing susceptible individuals to nephrolithiasis (28).

Although VC concentrations in muscle and bone are relatively low, their substantial mass renders them major reservoirs of VC in the body, with skeletal muscle alone accounting for 40%–67% of the total body VC pool (29, 30). SVCT2 is widely expressed in skeletal muscle (31), bone (32), and cartilage (33), serving as the primary transporter mediating VC uptake in these tissues. In the musculoskeletal system, VC contributes to key physiological processes, including muscle development and regeneration, as well as bone formation and remodeling (34, 35). Conversely, VC deficiency is associated with muscle atrophy, bone loss, and cartilage degeneration, suggesting a role in the initiation and progression of musculoskeletal disorders (36–38). A clearer understanding of VC function in musculoskeletal physiology and pathology may therefore help define its potential value in disease prevention and management.

## 4 Role of VC in musculoskeletal physiology

VC modulates multiple musculoskeletal cell types, including myoblasts, muscle stem cells (MuSCs), bone marrow mesenchymal stem cells (BMSCs), osteoblasts, osteocytes, osteoclasts, and chondrocytes. Its cell-specific effects and associated gene regulation are summarized in **Table 1**.

**Table 1:** Biological functions of VC in the musculoskeletal system.

Cell type	Related genes	Biological functions	Ref
Myoblast	• MyoD, MyoG, Myf5, Desmin, MyHC1/2/4/7, CKM, PCNA, MuRF1, MAFbx, Myostatin	Promotes MRF expression, myoblast proliferation and differentiation via antioxidant-dependent and -independent pathways, and may serve as a context-dependent synergistic enhancer of myogenesis.	(34, 43–49)
	• KDM7A, TET1/2, 5hmC, H3K9me1/2/3, H3K27me2/3	Epigenetically promotes muscle development.	(50–53)
MuSC	• Pax7, MyoD, MyoG, MyHC, CKM, Myf6, Cdk1/2, PCNA	Enhances MuSC proliferation, myogenic differentiation, and pool expansion, thereby promoting muscle repair and regeneration.	(56, 57, 59, 60)
BMSC	• ALP, Bglap2, Dlx3, COL22A1, Phospho1, SOST, DMP1, BMP-2, OCN, COL-I	Maintains BMSC phenotype and function while promoting their proliferation, migration, and osteogenic differentiation.	(64–67, 72–75)
	• KDM4B, TET2, 5hmC, H3K9me3/H3K27me3, miRNAs (miR-141, miR-200a)	Epigenetically programs osteochondrogenic differentiation.	(68–71)
Osteoblast	• ALP, OCN, COL-I, Bglap2, COL3A1, Runx2, Osx, BSP, Ihh, OPN, OPG, Phospho1	Promotes osteoblast proliferation and differentiation.	(76–84)
	• TET2, 5hmC	Epigenetically enhances osteogenic gene expression.	(68)
Osteocyte	• PHEX, DMP1, SOST, E11/gp38, Cd44, MMP2, MMP13	Promotes osteocyte formation and maturation.	(89–91)
Osteoclast	• RANKL, CTX, OPG	Bidirectionally modulates osteoclastogenesis.	(92–98)
Chondrocyte	• COL2A1, COL1A1, ACAN, SOX9, COMP, COL10A1, Runx2, MMP2, MMP13, ALP	Promotes chondrocyte maturation and hypertrophy.	(100–104, 106)
	• PHD2, 5hmC	Epigenetically regulates chondrogenesis.	(105)

MyHC, myosin heavy chain; CKM, Muscle creatine kinase; PCNA, Proliferating cell nuclear antigen; Cdk, Cyclin-dependent kinase; Bglap2, Bone gamma-carboxyglutamate protein 2; Dlx3, Distal-less homeobox 3; DMP1, Dentin matrix acidic phosphoprotein 1; SOST, Sclerostin; Ihh, Indian hedgehog; OPG, osteoprotegerin; PHEX, Phosphate-regulating neutral endopeptidase, X-linked; CTX, C-terminal telopeptide of type I collagen.

### 4.1 Skeletal muscle

**Figure 1:**
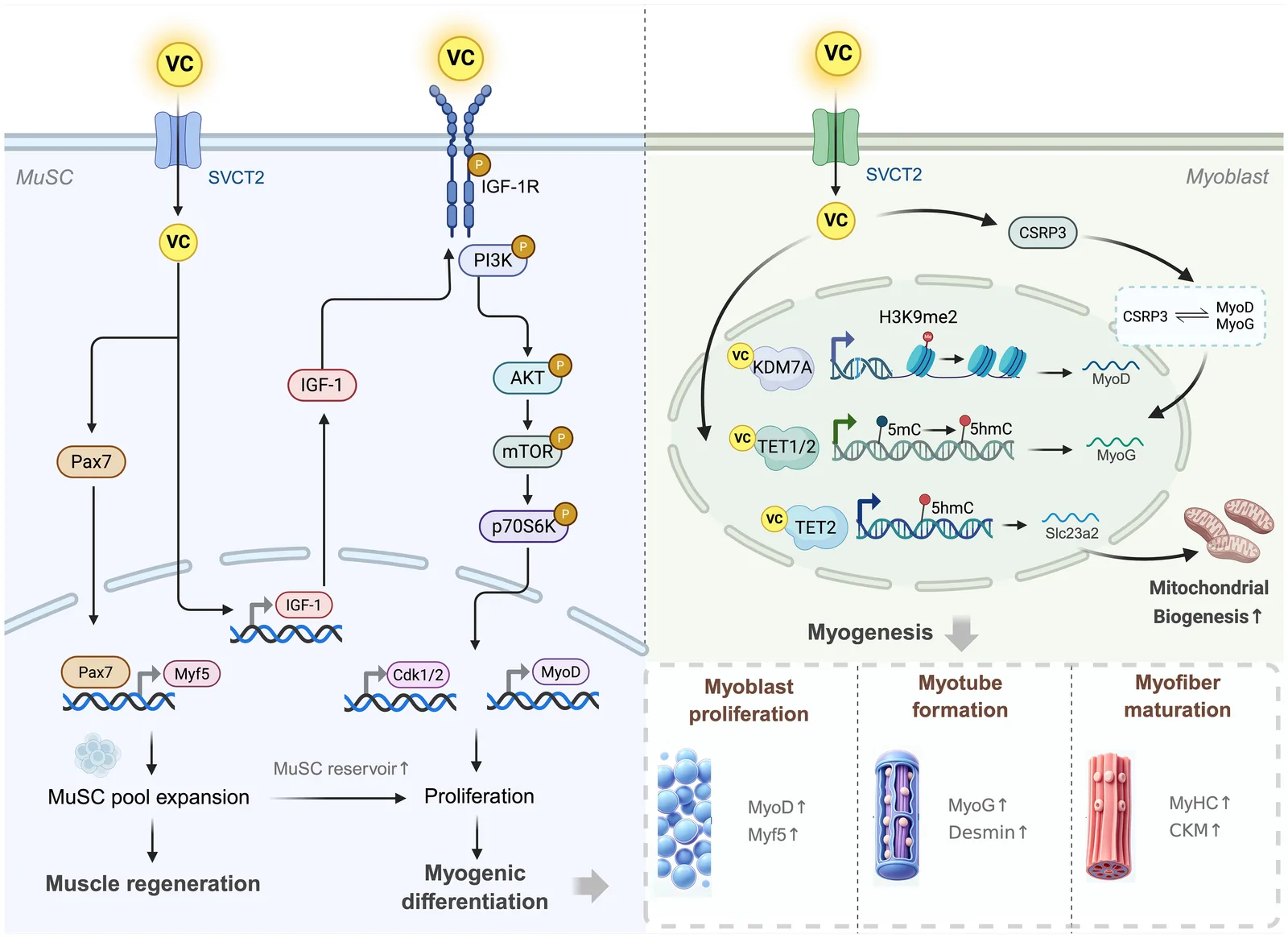
Potential regulatory roles of VC in muscle development and regeneration. VC promotes muscle regeneration by expanding the MuSC pool and enhances myogenic differentiation by stimulating MuSC proliferation. In myoblasts, VC upregulates the expression of MRFs, leading to cell proliferation and subsequent fusion into multinucleated myotubes, which ultimately mature into functional myofibers (Created with BioRender.com). Cdk, cyclin-dependent kinase; MyHC, myosin heavy chain.

Skeletal muscle constitutes approximately 40% of total body mass and is essential for locomotion, posture maintenance, and systemic metabolic regulation (39). Its basic structural unit is the myofiber, which is organized into fascicles surrounded by the perimysium; multiple fascicles together form skeletal muscle (40). Myofiber formation follows a coordinated sequence involving myogenic lineage commitment, myoblast proliferation, myotube fusion, and terminal maturation into functional myofibers (41). VC appears to regulate multiple stages of muscle development and contributes to post-injury repair and regeneration, thereby supporting skeletal muscle homeostasis (**Figure 1**).

#### 4.1.1 VC promotes muscle development

The complex process of skeletal muscle development is regulated by myogenic regulatory factors (MRFs), including myoblast determination protein 1 (MyoD), myogenic factor 5 (Myf5), and myogenin (MyoG) (42). VC can promote myogenesis by regulating MRF expression and activity through multiple mechanisms. In primary myoblasts derived from patients with OP, VC treatment alleviates ropivacaine-induced myotoxicity by reducing oxidative stress, thereby restoring MyoD and Myf5 expression and suppressing myostatin induction (43). Similarly, in pacu (*Piaractus mesopotamicus*) myoblasts, VC supplementation enhances MyoG expression and promotes cell proliferation and migration, partly by strengthening antioxidant defenses against menadione-induced oxidative injury (44). Beyond its antioxidant capacity, VC enhances myogenic differentiation in C2C12 myoblasts through cysteine and glycine-rich protein 3 (CSRP3)-dependent mechanisms, in which nuclear CSRP3 interacts with MyoD and MyoG during early and late differentiation, respectively (34). This CSRP3-mediated pathway also contributes to muscle regeneration in an animal model of muscle injury (34). Notably, the promyogenic effects of VC are context-dependent and may be amplified by mild cellular stress or growth-factor signaling. For example, capsaicin-induced mild endoplasmic reticulum stress enhances VC-mediated C2C12 differentiation (45), whereas inhibition of endogenous extracellular signal-regulated kinase (ERK) activity or fibroblast growth factor signaling produces similar enhancement (46, 47). Under low-temperature conditions, VC also strengthens insulin-like growth factor-I (IGF-1)-induced MyoG expression and myotube formation (48). In artificial skeletal muscle constructs, VC combined with mild heat stimulation promotes myotube formation, skeletal muscle hypertrophy, and active tension generation (49). These findings suggest that VC may act as a context-dependent regulator of myogenesis.

As an essential cofactor for DNA and histone demethylases, VC also contributes to the epigenetic regulation of myogenesis (50, 51). During myogenic differentiation, VC promotes a chromatin state favorable for myogenic gene expression by reducing repressive histone H3 lysine 9/27 (H3K9/H3K27) methylation and increasing 5-hydroxymethylcytosine (5hmC). This process is mediated by α-ketoglutarate-dependent dioxygenases (αKGDDs), including Jumonji C domain-containing lysine demethylase 7A (KDM7A) and ten-eleven translocation (TET) 1/2 DNA demethylases. KDM7A activates MyoD by removing H3K9me2 from its promoter, whereas TET1/2 promotes MyoG expression through 5hmC generation. Inhibition of αKGDDs by L-2-HG or knockdown of Kdm7a or Tet1/2 eliminates VC-induced myogenesis, demonstrating the requirement for its cofactor activity (52). VC also exerts intergenerational effects on skeletal muscle development. In mice, gestational VC supplementation upregulates TET2 in myogenic cells, promoting Slc23a2 demethylation and SVCT2 expression. This epigenetic regulation enhances mitochondrial biogenesis and oxidative myofiber development in offspring skeletal muscle, thereby improving endurance performance (53). Collectively, these findings suggest that VC promotes myogenesis through multiple biological mechanisms, highlighting its role in skeletal muscle development.

#### 4.1.2 VC regulates muscle regeneration by modulating MuSC fate

MuSCs, also known as satellite cells, are essential for skeletal muscle growth, repair, and regeneration. Located beneath the basal lamina of mature myofibers, MuSCs remain quiescent under homeostatic conditions but become activated after muscle injury, leading to proliferation and differentiation into multinucleated myotubes (54, 55). Emerging evidence suggests that VC regulates MuSC function and maintenance. VC enhances MuSC proliferation and differentiation by accelerating cell cycle progression and upregulating MRF expression, an effect mediated partly via the mechanistic target of rapamycin (mTOR) pathway (56). Further studies have refined this mechanism, implicating the phosphoinositide 3-kinase (PI3K)/protein kinase B (Akt)/mTOR/p70 ribosomal S6 kinase (p70S6K) signaling pathway, with SVCT2 and IGF-1 acting as key mediators. Inhibition of IGF-1 receptor (IGF-1R) or SVCT2 attenuates VC-induced pathway activation and myogenic effects, suggesting that VC may act through SVCT2-dependent uptake and IGF-1/IGF-1R-related signaling (57). VC also upregulates paired box protein 7 (Pax7) (56, 57), a master regulator of MuSC quiescence, differentiation, and long-term regenerative capacity (58). Recent evidence further identifies Pax7 as a direct molecular target of VC: VC binding enhances Pax7 expression and nuclear translocation, leading to Myf5 upregulation and C2C12 cell proliferation *in vitro*. In mouse models, VC-mediated Pax7 activation increases the MuSC population, promotes skeletal muscle regeneration after injury, and facilitates recovery from starvation-induced muscle atrophy (59). Moreover, VC treatment improves the functional capacity of MuSCs during prolonged *in vitro* culture by reducing oxidative stress and increasing ATP levels. Three-dimensional muscle tissues engineered from VC-treated MuSCs exhibit improved structural compaction, tensile strength, and amino acid content, suggesting potential utility of VC in MuSC-based tissue engineering strategies (60).

### 4.2 Bone formation and remodeling

**Figure 2:**
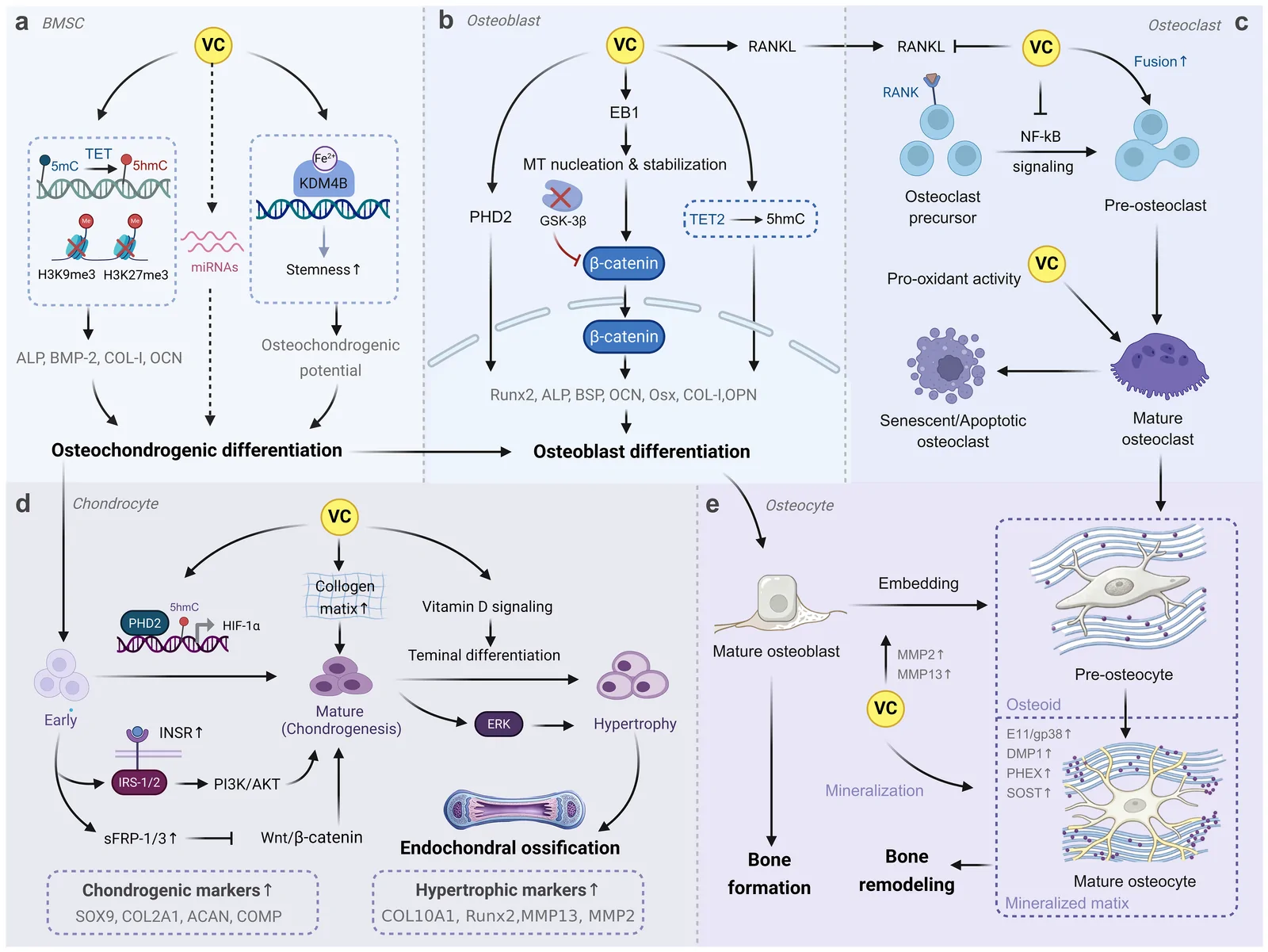
Potential regulatory roles of VC in bone formation and remodeling across different cell types. **(a)** VC promotes the osteochondrogenic differentiation of BMSCs through epigenetic mechanisms. **(b)** VC upregulates the expression of osteogenic markers, thereby promoting osteoblast differentiation and maturation. **(c)** VC suppresses osteoclastogenesis while promoting the fusion and differentiation of pre-osteoclasts. Subsequently, mature osteoclasts either undergo senescence/apoptosis or attach to the bone surface to perform resorption. **(d)** VC promotes early chondrocyte maturation (chondrogenesis) and late-stage hypertrophy differentiation (endochondral ossification). **(e)** VC-stimulated mature osteoblasts participate in bone formation, with approximately 5–20% becoming progressively embedded within the surrounding osteoid. VC further facilitates osteocytogenesis by promoting osteoid mineralization and the expression of osteocyte-specific markers, thereby contributing to the regulation of bone formation and remodeling (Created with BioRender.com). MT, microtubule; GSK-3β, glycogen synthase kinase-3β; sFRP-1/3, secreted Frizzled-related proteins 1 and 3; DMP1, dentin matrix protein 1; PHEX, phosphate-regulating endopeptidase homolog X-linked; SOST, sclerostin.

Bone is a highly specialized and dynamic connective tissue that supports muscles and underpins mobility (61). Bone homeostasis depends on the balance between osteoblast-mediated bone formation and osteoclast-mediated bone resorption (2). VC is closely linked to bone formation and metabolism (10, 62), whereas inadequate VC status is associated with compromised bone structure, reduced strength, impaired growth, and delayed healing (11). This section summarizes the roles of VC in major bone-resident cell populations (**Figure 2**).

#### 4.2.1 BMSCs

BMSCs are multipotent mesenchymal cells capable of differentiating into osteogenic, chondrogenic, and adipogenic lineages, making them essential for skeletal homeostasis and tissue regeneration (63). VC supports multiple aspects of BMSC biology, including phenotypic maintenance, proliferation, migration, osteogenic differentiation, and extracellular matrix (ECM) production (64–66). It also upregulates osteogenic markers, including alkaline phosphatase (ALP), bone morphogenetic protein-2 (BMP-2), type I collagen (COL-I), and osteocalcin (OCN) (67). Evidence from human donor-derived BMSCs suggests that VC is required for ECM deposition, mineralization, and osteogenic gene induction during osteogenic differentiation. Even under adipogenic induction conditions, VC can redirect BMSCs toward an osteogenic transcriptional program, indicating a strong influence on lineage commitment. A key mechanism underlying these effects is VC-dependent epigenetic remodeling. By regulating histone demethylation, particularly H3K9me3 and H3K27me3, and promoting TET-mediated 5hmC accumulation at promoters and distal regulatory elements near bone-related genes, VC establishes a transcriptionally permissive epigenetic landscape for osteogenesis (68). VC also promotes the specification, long-term self-renewal, and osteochondrogenic potential of human pluripotent stem cell-derived BMSCs *in vitro* through the iron-dependent histone demethylase KDM4B (69). These findings suggest that epigenetic regulation is a central mechanism linking VC to BMSC-mediated osteogenesis. In addition, microRNA (miRNA) profiling indicates that VC modulates miRNA expression during BMSC proliferation and osteogenic differentiation (70). In mouse BMSCs, miR-141 and miR-200a repress SVCT2 expression and attenuate osteogenic differentiation, whereas inhibition of these miRNAs enhances SVCT2 and osteogenic marker expression (71). Thus, miRNA-mediated regulation of SVCT2 may further shape VC responsiveness during BMSC osteogenesis. VC also has potential utility in BMSC-based bone tissue engineering. VC-functionalized poly(methyl methacrylate), combined with soluble VC, enhances the osteogenic differentiation of human BMSCs and mitigates the decline in osteogenic potential during prolonged *in vitro* expansion (72). VC-containing mineralized collagen scaffolds increase BMSC metabolic activity, ALP activity, and mineral deposition, while delaying expression of genes associated with osteoclast activity and bone resorption (73). In mouse implantation models, sustained-release poly(D,L-lactide-co-glycolide) scaffolds containing biologically active VC and dexamethasone support BMSC-mediated mineralized bone formation through intramembranous ossification (74, 75). Collectively, these findings suggest that VC promotes BMSC osteogenic differentiation and may improve BMSC-based bone tissue engineering strategies, although its precise molecular targets require further clarification.

#### 4.2.2 Osteoblasts

VC contributes to osteoblast proliferation and differentiation (76–78). VC-deficient mouse models show osteopenia, spontaneous fractures, and dysplastic proliferation of immature osteoblasts (79), phenotypes associated with impaired osteoblast differentiation and a shift toward peroxisome proliferator-activated receptor γ-dependent adipogenesis (80). VC supplementation restores osteoblast differentiation, prevents dysplastic proliferation, and normalizes body weight, bone mass, bone mineral content, and bone mineral density (BMD) in VC-deficient mice (79, 81). Beyond correcting developmental defects, VC also attenuates osteoblast senescence by expanding the pool of proliferative cells (82). At the molecular level, VC promotes osteoblast differentiation partly by inducing osterix (Osx), a key transcription factor for osteoblast maturation. This process involves activation of prolyl hydroxylase domain (PHD) enzymes, which promote proteasomal degradation of transcriptional repressors of the Osx gene (83). VC also regulates osteogenic signaling through end-binding protein 1 (EB1), a conserved microtubule plus-end tracking protein. By enhancing EB1 expression and microtubule organization, VC stabilizes β-catenin at cell–cell contacts and limits glycogen synthase kinase-3β-mediated degradation, thereby supporting runt-related transcription factor 2 (Runx2)-dependent expression of early osteogenic markers, including ALP, bone sialoprotein (BSP), and OCN (84). Epigenetic regulation represents another mechanism for VC-mediated osteoblast differentiation. Through TET2-dependent 5hmC accumulation, VC promotes an open chromatin state that supports coordinated expression of pro-osteogenic genes (68).

#### 4.2.3 Osteocytes

Osteocytes, the most abundant cells in bone, are terminally differentiated, non-proliferative osteoblast-derived cells that play crucial roles in regulating bone formation and remodeling. During bone formation, mature osteoblasts become progressively embedded within the COL-I-rich osteoid, pass through a pre-osteocyte stage, and ultimately mature as the surrounding matrix mineralizes (85). VC contributes to mineralization and osteocytogenesis (86–88). In the IDG-SW3 osteoblast-to-osteocyte cell line, VC deprivation reduces collagen production, decreases ALP activity, and severely impairs mineralization (89). Similarly, in MLO-A5 pre-osteocyte cultures, VC promotes progressive ECM mineralization and increases the expression of osteocyte-specific markers, including E11/gp38 and sclerostin (90). In addition, VC modulates matrix metalloproteinases (MMPs), including MMP2 and MMP13, thereby facilitating collagenous ECM remodeling, osteocyte embedding, and subsequent mineralization (91).

#### 4.2.4 Osteoclasts

Osteoclasts are multinucleated bone-resorbing cells that play a central role in bone remodeling. VC appears to regulate osteoclastogenesis in a context-dependent manner. VC deficiency enhances osteoclast formation and bone resorption (92, 93), whereas VC supplementation can inhibit the receptor activator of nuclear factor κB ligand (RANKL)-induced differentiation of osteoclast precursors into mature osteoclasts (94). Under pathological conditions, such as a high-cholesterol diet, VC suppresses osteoclastogenesis by downregulating RANKL expression and nuclear factor kappa B (NF-κB) signaling (95). However, VC may also indirectly promote osteoclast formation by modifying the bone microenvironment. By stimulating BMSC-derived ECM production, particularly collagen, VC contributes to the formation of a matrix niche that enhances RANKL expression in stromal cells and facilitates the generation of tartrate-resistant acid phosphatase (TRAP)-positive osteoclasts (96). VC may also exert direct cytotoxic effects on mature osteoclasts through pro-oxidant activity, while indirectly enhancing osteoclastogenesis via RANKL upregulation in osteoblasts (97). These findings suggest that VC exerts stage-dependent effects on osteoclastogenesis, supporting precursor fusion and differentiation at early stages while promoting senescence or apoptosis of mature osteoclasts at later stages (98). Collectively, VC may regulate bone homeostasis by modulating osteoclastogenesis in a context- and stage-dependent manner.

#### 4.2.5 Chondrocytes

Chondrocytes, the resident cells of cartilage, synthesize and maintain the cartilaginous ECM and play an essential role in endochondral ossification (99). Cartilage accumulates relatively high levels of VC, which is associated with SVCT2 expression in chondrocytes (33). VC promotes chondrocyte maturation and subsequent hypertrophy by upregulating cartilage matrix genes, including SRY-box transcription factor 9 (SOX9), COL2A1, aggrecan (ACAN), and cartilage oligomeric matrix protein (COMP), as well as hypertrophy-related markers such as COL10A1, Runx2, MMP13, and MMP2, thereby facilitating endochondral ossification (100–102). Mechanistically, VC promotes early chondrocyte differentiation by facilitating collagenous matrix formation, which activates the ERK signaling pathway to drive further differentiation (103). VC also enhances terminal chondrocyte differentiation by promoting 1,25-dihydroxyvitamin D_3_ synthesis and upregulating the vitamin D receptor, thereby potentiating vitamin D genomic signaling (104). Beyond endochondral ossification, VC contributes to cartilage development and homeostasis. In mice, VC deficiency reduces articular cartilage area and width. In primary mouse articular chondrocytes, VC promotes proliferation and maturation partly through a PHD2-dependent increase in 5hmC at the promoter region of hypoxia-inducible factor 1α (HIF-1α), a key differentiation-related gene (105). Additional evidence from ATDC5 chondrogenic cells suggests that VC promotes chondrogenic differentiation through coordinated regulation of PI3K/Akt and Wnt/β-catenin signaling. Specifically, VC activates PI3K/Akt signaling by upregulating the insulin receptor (INSR) and its substrates IRS-1 and IRS-2, while reversing insulin-induced feedback inhibition of IRS-1/2. Concurrently, VC increases secreted Frizzled-related proteins 1 and 3, thereby inhibiting Wnt/β-catenin signaling and relieving its suppressive effect on chondrogenic differentiation (106). Collectively, these findings suggest that VC promotes chondrocyte differentiation, maturation, and hypertrophy, thereby contributing to chondrogenesis and endochondral bone formation.

### 4.3 Tendon–bone repair

In the musculoskeletal system, tendons transmit force and bones provide load-bearing support; the coordinated healing of these tissues is essential for restoring motor function (107, 108). Given its antioxidant properties and role in collagen synthesis, VC may contribute to tendon and bone repair. **Table 2** summarizes preclinical evidence for the role of VC in tendon–bone repair.

**Table 2:** Preclinical evidence for the role of VC in tendon–bone repair.

Study type	Dosage and administration of VC	Target cell/tissue	Mechanism	Ref
Tendon repair				
*In vitro*	L-ascorbic acid 2-phosphate, 50 μg/mL	Rabbit Achilles tendon tenocytes	• COL-I, MKX, DCN↑	(115)
*In vivo*	Intraperitoneal injection of VC, 150 mg every 2 days	Achilles tendon rupture repair model in Wistar rats	• Angiogenesis↑ • COL-I↑	(116)
*In vivo*	Local (paratendinous) injection of VC, 30 mM every 2 days	Achilles tendon rupture repair model in Wistar rats	• Achilles functional index↑	(117)
*In vivo*	VC (sodium ascorbate), 1% (wt/vol) in drinking water	Infraspinatus tendon rupture repair model in SD rats	• COL-III, SOD1↑ • MMP13↓ • Oxidative damage↓	(118)
*In vitro*	VC, 150 μM or 30 mM	H_2_O_2_-treated human tenocytes	• Cell viability↑ • COL-I↑ • ROS, P16↓	(123)
*In vitro*	Tendoactive^®^ (VC-containing nutraceutical)	IL-1β-treated primary human tenocytes	• COL-I, β1-integrin↑ • NF-κB, p65, COX-2, MMP1, caspase-3↓	(124)
Bone repair				
*In vivo*	VC-supplemented drinking water, 330 mg/L	Spontaneous fracture mice	• ALP, OCN, COL-I↑	(128)
*In vitro*	L-ascorbic acid 2-phosphate, 300 μg/mL	BMSCs and osteoblasts derived from mice		
*In vivo*	VC-supplemented drinking water, 1 mg/mL	Osteogenic disorder Shionogi rats	• OCN, MGP, OPN↑	(129)
*In vivo*	Intraperitoneal injection of VC, 200 mg/kg	Nicotine-induced tibial defect model in SD rats	• BMP-2↑ • HIF-1α↓	(131)
*In vitro*	25 wt% VC-incorporated polycaprolactone membranes	Human fetal osteoblasts	• ALP, COL-I, OPN↑ • ROS↓	(134)
*In vitro*	25 wt% VC-incorporated polycaprolactone membranes	Co-culture system of human fetal osteoblast and THP-1–derived osteoclast	• ALP, OCN, COL-I↑ • Mineralization↑ • RANKL/OPG, ROS↓	(135)
*In vitro*	VC-conditioned pure Ti and double-etched Ti surfaces using 100 mM VC saline	MC3T3-E1 osteoblasts	• Runx2, Osx, OCN↑	(136)
*In vitro*	Hydroxyapatite-coated Ti6Al4V discs incorporating 500 μg VC	Human fetal osteoblasts and THP-1 monocytes (osteoclast precursor)	• Osteoblast proliferation↑ • Antibacterial activity↑ • Osteoclastogenesis↓	(137)
*In vitro*	Polydopamine nanospheres reduced using 300 mM VC	MC3T3-E1 osteoblasts	• ALP↑ • H_2_O_2_ scavenging capacity↑	(138)
*In vivo*	VC/simvastatin acid co-loaded injectable hydrogel	Critical-sized calvarial defect model in aged rats	• ALP↑ • Bone defect healing↑	(139)
*In vivo*	L-ascorbic acid 2-phosphate (250 mM)-containing multicomponent scaffold	Critical-sized calvarial defect model in aged rats	• OCN↑ • Bone defect healing↑	(140)

MKX, Mohawk; DCN, decorin; COX-2, cyclooxygenase-2; MGP, Matrix Gla protein; OPG, osteoprotegerin.

↑ indicates an increase; ↓ indicates a decrease.

#### 4.3.1 Tendon repair

Tendons are mechanically sensitive, COL-I-rich tissues that connect muscle to bone and transmit high loads during movement. Tenocytes regulate cellular metabolism and ECM turnover, thereby maintaining tendon structural integrity and functional adaptation (109, 110). VC appears to support tendon biology by promoting tenocyte proliferation, enhancing mesenchymal stem cell tenogenic differentiation, and preserving tendon stem cell viability, migration, stemness, and tenogenic potential (111–114). At the molecular level, VC upregulates tendon-related genes, including COL-I, COL-III, Mohawk, decorin, and scleraxis, while enhancing collagen-rich ECM deposition (112, 115).

Preclinical evidence suggests that VC may facilitate tendon repair. In rat Achilles tendon rupture models, high-dose VC supplementation enhances early angiogenesis and COL-I synthesis, followed by improved connective tissue organization and increased collagen fibril diameter during later healing stages (116). VC administration also reduces functional impairment after tenotomy in rats (117). In a rat rotator cuff repair model, VC treatment improves early chondrogenic and antioxidant responses and later enhances collagen fiber formation and fibrocartilage area, accompanied by reduced oxidative damage and favorable ECM remodeling (118). VC also attenuates rotator cuff degeneration in SOD1-deficient mice, supporting a protective role under redox-imbalanced conditions (119). In tendon injury models, VC combined with bioactive scaffold materials, including marine collagen (120), hyaluronic acid (121), and fibrin clot (122), shows potential as a bioactive adjunct in tendon repair strategies. Mechanistically, VC appears to act partly through antioxidant and anti-inflammatory pathways. In H_2_O_2_-treated human tenocytes, high-dose VC (30 mM) suppresses reactive oxygen species (ROS) production, protects the cytoskeleton from oxidative damage, preserves mitochondrial function and ATP production, and reduces p16 expression (123). In interleukin-1β (IL-1β)-stimulated primary human tenocytes, a VC-containing nutraceutical inhibits NF-κB activation and p65 nuclear translocation, thereby downregulating catabolic markers and upregulating anabolic markers such as COL-I and β1-integrin (124). Clinical evidence remains limited. In patients undergoing rotator cuff repair, postoperative oral VC supplementation (500 mg/day for 45 days) is associated with a lower tendon non-healing rate at 6-month follow-up compared with non-supplemented controls (11% vs. 23%) (125). However, the available evidence remains insufficient to establish the clinical efficacy of VC supplementation for tendon repair.

#### 4.3.2 Bone repair

Bone repair and regeneration are complex biological processes, yet current evidence supporting a beneficial role of VC in these processes remains largely preclinical, with limited clinical validation (126, 127). In spontaneous fracture mice carrying GULO mutations with impaired VC biosynthesis, VC supplementation restores osteoblast differentiation and mineralized nodule formation, supporting adequate VC availability for bone regeneration (128). Similarly, in osteogenic disorder Shionogi rats with impaired VC synthesis, VC deficiency suppresses collagen synthesis and reduces OCN-, matrix Gla protein-, and osteopontin (OPN)-expressing cells during fracture repair, whereas VC supplementation rapidly induces callus formation and restores OPN expression in differentiated osteoblasts and hypertrophic chondrocytes (129). However, these effects are less consistent in models without pre-existing VC deficiency. In normal rats, VC supplementation does not accelerate healing after experimental tibial fracture (130). In a nicotine-induced tibial defect model, VC alone does not significantly promote bone healing, although co-administration with nicotine attenuates bone necrosis and improves new bone formation, possibly by counteracting nicotine-related molecular changes (131). These findings suggest that VC may be more effective for bone repair under conditions of VC deficiency or impaired bone healing than under normal physiological conditions. In terms of clinical evidence, a randomized placebo-controlled trial in 336 adults with distal radial fractures shows that oral VC supplementation (500 mg/day for 50 days) does not improve functional outcomes or fracture healing compared with placebo (132). In patients undergoing long-bone fixation, a one-week course of antioxidant tablets (vitamins A, C, and E and selenium) reduces lipid peroxidation and increases SOD and glutathione reductase activities, followed by elevated serum OCN and ALP levels at 2 weeks (133). However, baseline VC status is not reported, and the observed effects likely reflect the combined action of multiple antioxidants rather than VC alone.

Despite these uncertainties, VC has attracted increasing attention as a bioactive component in bone tissue-engineering strategies. *In vitro* evidence indicates that VC-incorporated polycaprolactone membranes reduce oxidative stress and upregulate osteogenic genes, including ALP, COL-I, and OPN, in human fetal osteoblasts (134). In osteoblast–osteoclast co-culture systems, these membranes promote an osteogenic microenvironment, characterized by a reduced RANKL/osteoprotegerin ratio, increased bone formation markers, and enhanced ECM mineralization (135). VC-based surface modification of titanium implants and hydroxyapatite coatings also improves osteoblast viability, adhesion, proliferation, osteogenic protein expression, and antibacterial activity, while inhibiting osteoclast differentiation *in vitro* (136, 137). Advanced VC-based biomaterials further support its potential role in experimental bone regeneration. VC-reduced polydopamine nanoparticle-functionalized nanofibrous scaffolds exhibit enhanced H_2_O_2_-scavenging capacity and promote BMP-2-induced osteogenic differentiation *in vitro* (138). In aged rat models, injectable hydrogels co-loaded with VC and simvastatin acid promote new bone formation and defect closure (139). Multicomponent scaffolds incorporating VC, fibroblast growth factor-2, and human adipose-derived stem cells also enhance stem cell stemness, multilineage differentiation, and repair of critical-sized bone defects in aged rats (140). Collectively, these preclinical findings suggest that VC may serve as a promising bioactive component for bone tissue-engineering strategies, although clinical validation remains lacking.

## 5 Preclinical mechanisms underlying the role of VC in musculoskeletal disorders

**Figure 3:**
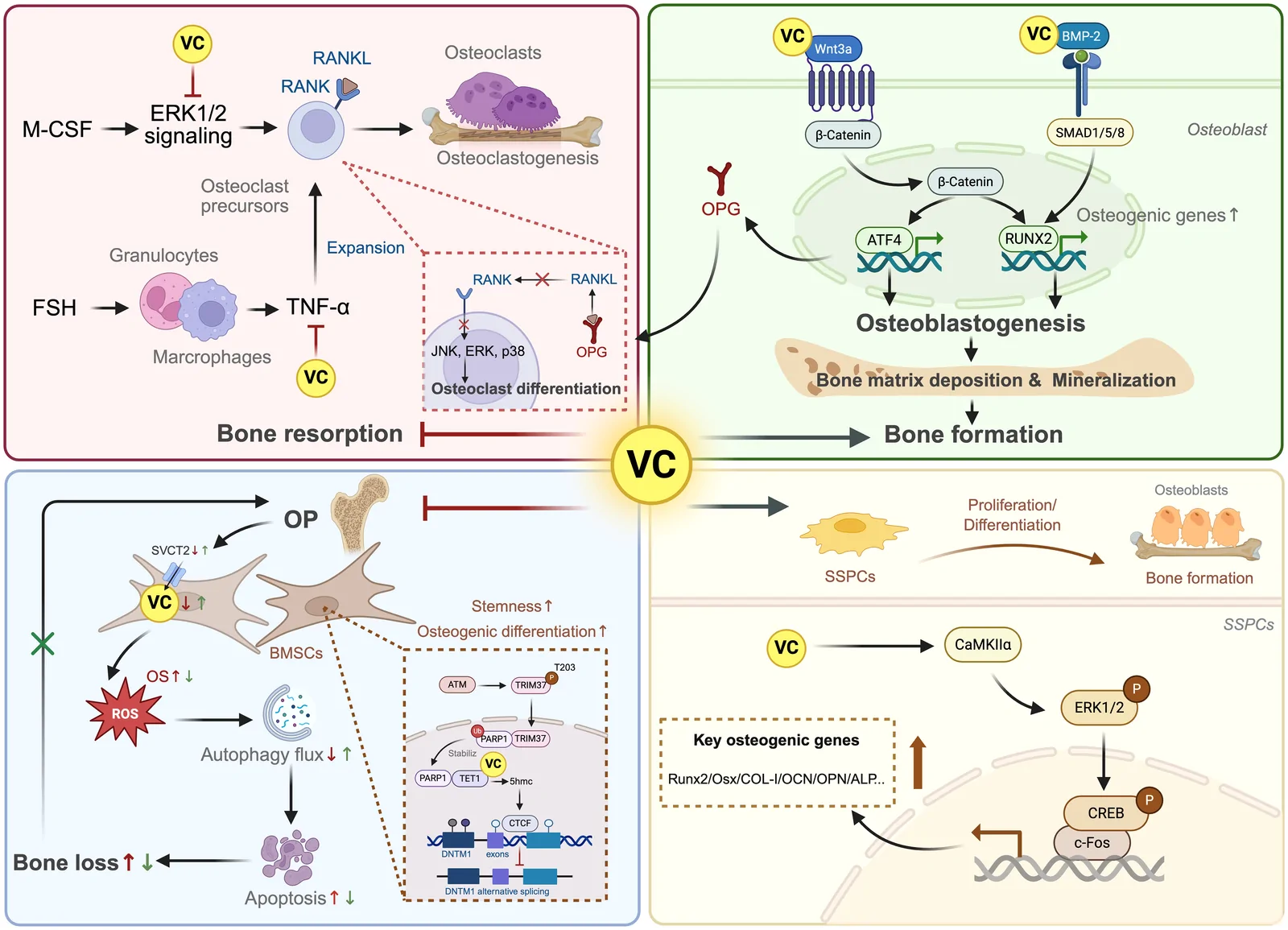
Potential regulatory mechanisms of VC in OP. VC promotes osteoblastogenesis while suppressing osteoclastogenesis, thereby enhancing bone formation and reducing bone resorption. Furthermore, VC stimulates the osteogenic differentiation of BMSCs and skeletal stem and progenitor cells (SSPCs), alleviating bone loss and facilitating new bone formation (Created with BioRender.com). M-CSF, macrophage colony-stimulating factor; FSH, follicle-stimulating hormone; OPG, osteoprotegerin; SMAD, suppressor of mothers against decapentaplegic.

**Figure 4:**
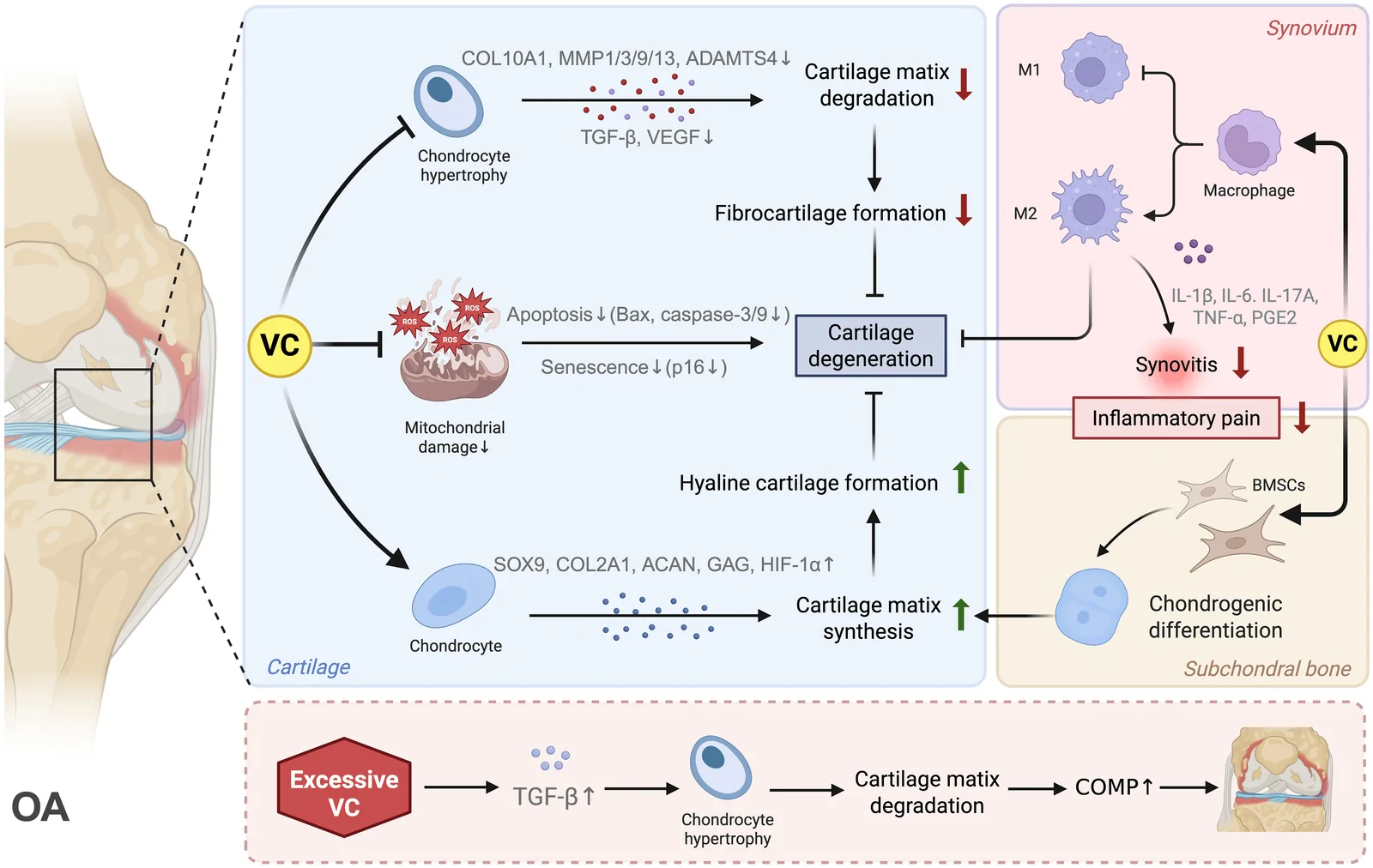
Potential regulatory mechanisms of VC in OA. In cartilage, VC promotes hyaline cartilage regeneration by enhancing cartilage matrix synthesis, while suppressing chondrocyte hypertrophy to reduce matrix degradation and fibrocartilage formation. Additionally, VC attenuates mitochondrial oxidative damage, thereby alleviating chondrocyte apoptosis and senescence. These mechanisms collectively mitigate cartilage degeneration. In the synovium, VC promotes M2 polarization of synovial macrophages, thereby reducing synovial inflammation and pain. In subchondral bone, VC facilitates chondrogenic differentiation of BMSCs, further attenuating cartilage degeneration. However, excessive VC may accelerate cartilage matrix degradation and exacerbate OA progression (Created with BioRender.com). ADAMTS4, a disintegrin and metalloproteinase with thrombospondin motifs 4; VEGF, vascular endothelial growth factor; BAX, BCL2-associated X, apoptosis regulator; GAG, glycosaminoglycan; PGE2, prostaglandin E2.

**Figure 5:**
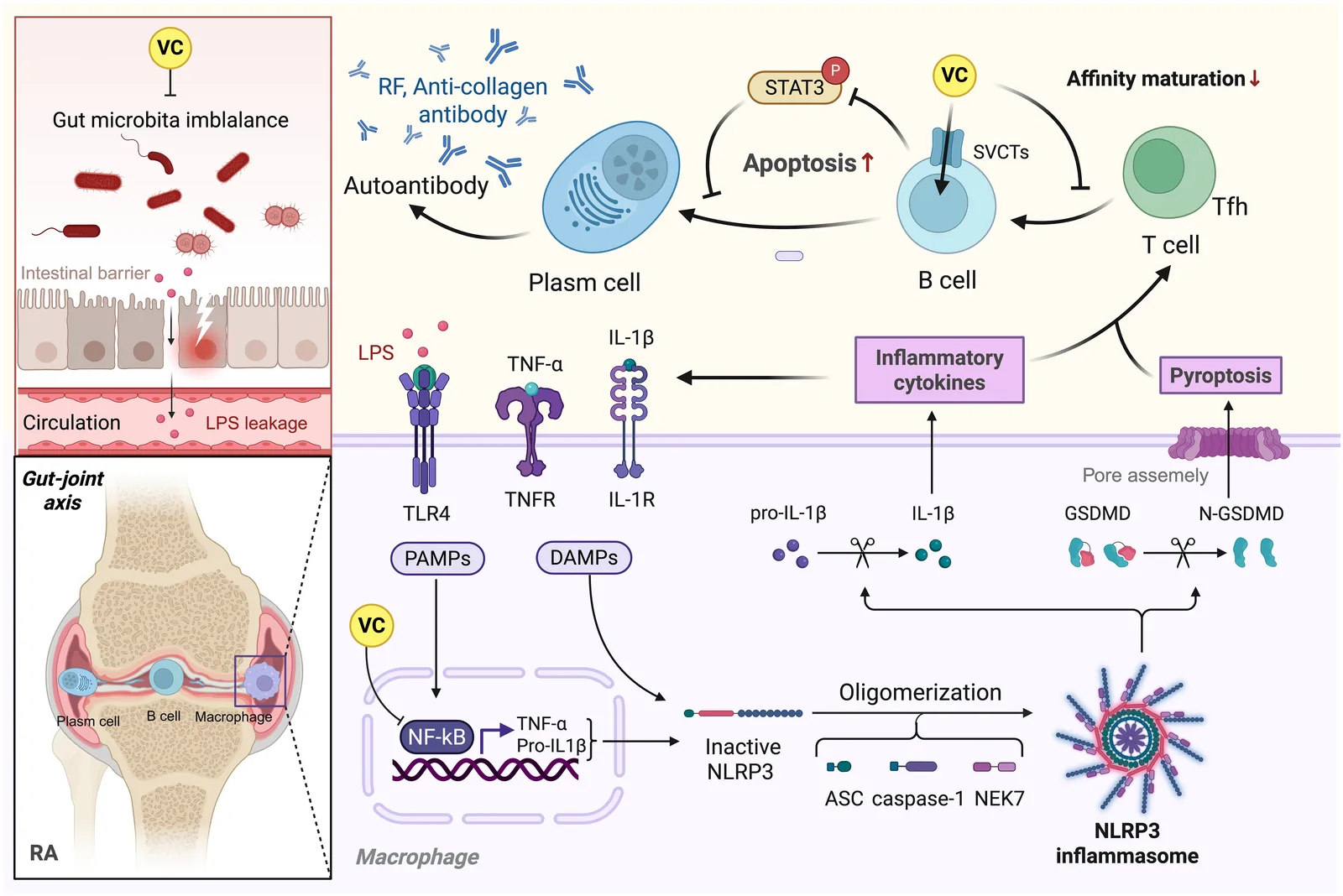
Potential regulatory mechanisms of VC in RA. RA progression is associated with gut microbiota imbalance and intestinal barrier disruption, leading to bacterial translocation and increased circulating lipopolysaccharide (LPS) levels. Pathogen-associated molecular patterns (PAMPs), such as LPS, activate the NF-κB signaling pathway, promoting inflammatory cytokine production and subsequent NLRP3 inflammasome activation, which induces macrophage inflammation and pyroptosis. Pyroptosis-associated release of damage-associated molecular patterns (DAMPs), such as TNF-α and IL-1β, further amplifies inflammatory responses and creates a vicious cycle. In addition, these processes promote immune activation and autoantibody production. VC attenuates RA progression by restoring gut microbiota balance, alleviating intestinal barrier injury, and suppressing NF-κB/NLRP3-mediated macrophage inflammation and pyroptosis. Additionally, VC intrinsically inhibits B-cell differentiation into plasma cells and impairs antibody affinity maturation, thereby reducing autoantibody production (Created with BioRender.com).

**Figure 6:**
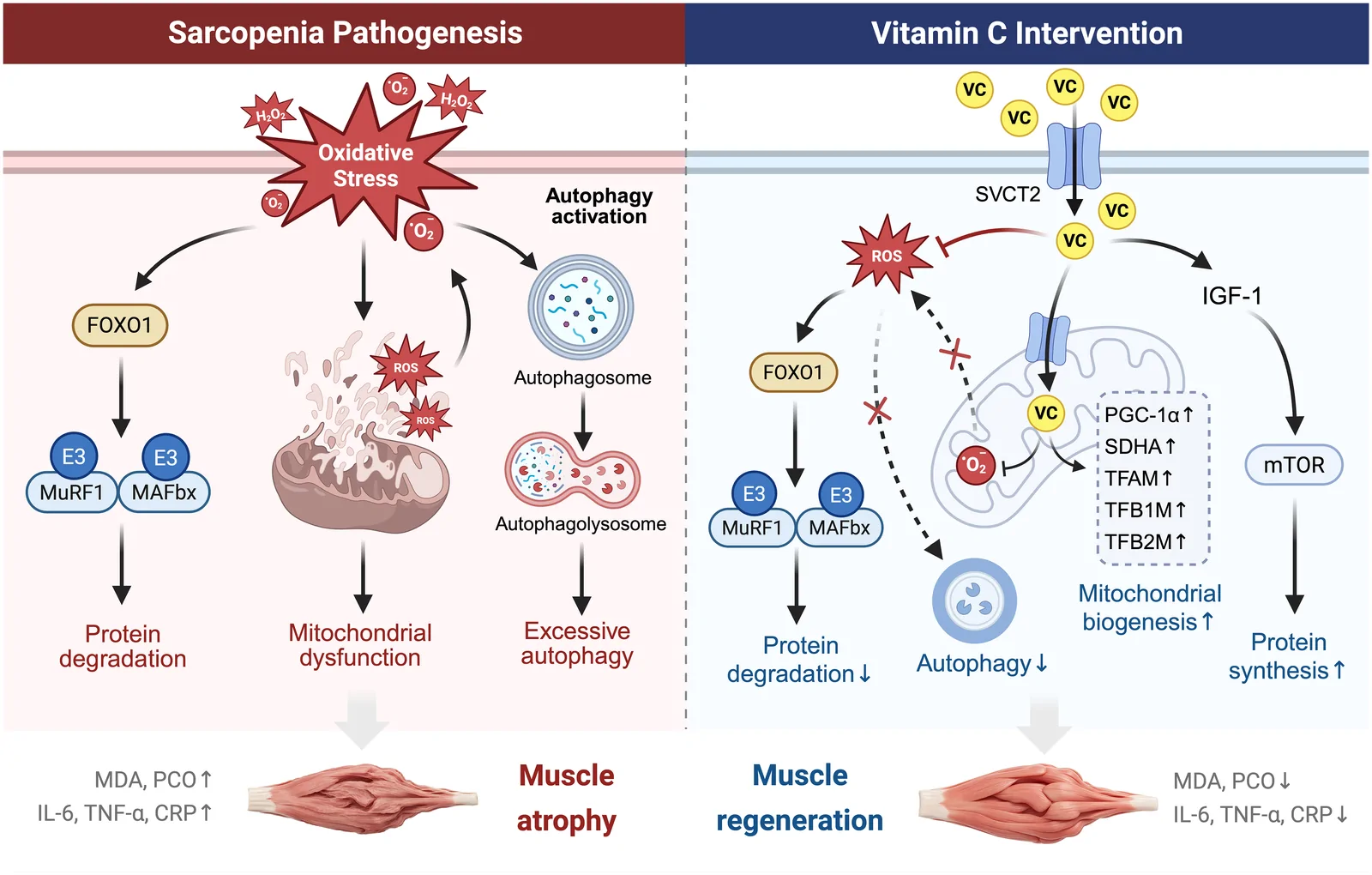
Potential regulatory mechanisms of VC in sarcopenia. Oxidative stress plays a central role in the pathogenesis of sarcopenia by activating the ubiquitin–proteasome system, inducing mitochondrial dysfunction, and triggering excessive autophagy, collectively leading to muscle atrophy. VC counteracts these pathological processes by attenuating oxidative stress, thereby inhibiting protein degradation and autophagy, while simultaneously enhancing protein synthesis and mitochondrial biogenesis, ultimately promoting muscle regeneration (Created with BioRender.com). PCO, protein carbonyl; PGC-1α, peroxisome proliferator-activated receptor gamma coactivator 1-alpha; SDHA, succinate dehydrogenase complex flavoprotein subunit A; TFAM, mitochondrial transcription factor A; TFB1M, mitochondrial transcription factor B1.

Musculoskeletal disorders severely affect bones, joints, and skeletal muscles, leading to impaired mobility and reduced quality of life. Given the close association between VC and musculoskeletal physiology, this section summarizes preclinical evidence from cellular and animal studies regarding the potential molecular mechanisms by which VC influences four major musculoskeletal disorders: OP (**Figure 3**), OA (**Figure 4**), RA (**Figure 5**), and sarcopenia (**Figure 6**). Detailed information on the related studies is provided in **Supplementary Table 1**.

### 5.1 OP

OP is an age-related metabolic bone disorder characterized by deterioration of bone microarchitecture, loss of bone mass, and increased fracture risk. Its pathogenesis is primarily driven by an imbalance between osteoclast-mediated bone resorption and osteoblast-mediated bone formation (2). Preclinical evidence suggests that VC participates in the regulation of this remodeling balance. *In vivo*, VC deficiency increases the number of TRAP-positive osteoclasts and enhances bone resorption (141), whereas VC supplementation reverses bone loss in osteoporotic rats (142). In ovariectomized (OVX) mice, VC supplementation increases lumbar spine and tibial BMD, improves trabecular microarchitecture, and enhances bone formation indices, including mineralizing surface, mineral apposition rate, bone formation rate, and plasma OCN levels (143). Mechanistically, VC appears to suppress osteoclastogenesis while promoting osteoblastogenesis. *In vitro*, VC inhibits osteoclast differentiation of osteoclast precursors by suppressing macrophage colony-stimulating factor-induced RANK expression through ERK1/2 signaling (144). In hypogonadal bone loss, elevated follicle-stimulating hormone may stimulate bone marrow granulocytes and macrophages to produce tumor necrosis factor-α (TNF-α), thereby expanding the osteoclast precursor pool; VC attenuates this TNF-α-mediated osteoclastogenic effect by promoting TNF-α catabolism (145). On the bone-forming side, BMSCs from VC-treated OVX mice show increased expression of osteogenic genes, including BSP, Osx, BMP-2, Runx2, and ALP, compared with those from non-supplemented mice (143). Magnesium ascorbyl phosphate, a stable VC derivative, promotes skeletal stem and progenitor cell proliferation and osteogenic differentiation by activating the calcium/calmodulin-dependent serine/threonine kinase IIα (CaMKIIα)/ERK1/2/cAMP-response element-binding protein (CREB)/c-Fos signaling pathway. *In vivo*, it prevents OVX-induced bone loss in mice and accelerates femoral bone defect healing in rats (35). Further evidence from OVX rats indicates that VC promotes osteoblastogenesis while suppressing osteoclastogenesis, an effect associated with activation of the Wnt3a/β-catenin/activating transcription factor 4 (ATF4) and mitogen-activated protein kinase (MAPK) signaling pathways (146).

SVCT2-mediated VC uptake also appears to be important for preserving bone cell function under osteoporotic conditions. In a type 1 diabetes-induced OP mouse model, SVCT2 expression is markedly reduced in bone tissue and bone marrow, accompanied by decreased antioxidant capacity and impaired bone quality (147). In BMSCs, SVCT2 knockdown reduces VC uptake, increases oxidative stress, impairs autophagic flux, and heightens susceptibility to apoptosis. VC supplementation protects normal BMSCs by attenuating oxidative stress and inducing protective autophagy, but these effects are lost when SVCT2 is knocked down (148). Thus, intact SVCT2 function appears necessary for VC-mediated BMSC protection and bone homeostasis. Epigenetic regulation further links VC to bone aging and OP. In BMSCs, VC promotes 5hmC accumulation at DNMT1 exons through activation of the TRIM37/PARP1/TET1 axis, facilitates DNA demethylation, and supports CTCF-mediated suppression of DNMT1 alternative splicing. This epigenetic program attenuates BMSC replicative senescence while enhancing self-renewal and osteogenic differentiation. In aged mice, activation of this pathway is associated with reduced OP-like bone loss (149). Notably, VC deficiency compromises bone microarchitecture and mineralization in adult GULO^−^/^−^ mice in a sex-specific manner, with females showing greater susceptibility than males (150). Although the underlying mechanisms remain unclear, this sex-specific vulnerability may be partly related to differences in VC requirement, baseline VC status, and proteomic responses to VC deficiency and re-supplementation (151–153). Overall, these preclinical findings suggest that VC modulates OP-related bone remodeling through multiple mechanisms. Further studies are needed to define its molecular targets and clarify the sex-specific mechanisms underlying its effects on bone.

### 5.2 OA

OA is the most prevalent degenerative joint disease and is characterized by progressive cartilage degradation, subchondral bone remodeling, synovial inflammation, osteophyte formation, and joint pain (154). Oxidative stress is one of the key mechanisms driving OA progression (155). In human chondrocytes, VC attenuates ROS-induced telomere shortening, replicative senescence, and impaired glycosaminoglycan synthesis (156). Its antioxidant effects in chondrocytes have been consistently reported (157, 158). VC also alleviates ATDC5 cell senescence by restoring mitochondrial dynamics and reducing mitochondrial superoxide production (159). Beyond antioxidant protection, VC modulates apoptosis-related proteins, suppresses pro-inflammatory cytokines, and reduces MMP expression in monosodium iodoacetate (MIA)-treated SW1353 chondrosarcoma cells. *In vivo*, VC treatment attenuates cartilage degeneration in MIA-induced OA rat models (160). Notably, the lowest dose (100 mg/kg) shows the greatest protective effect, whereas higher doses (200 and 300 mg/kg) provide no additional benefit and instead reduce this effect (160). Similarly, in a rabbit microfracture-induced cartilage defect model, intra-articular VC at 10 mg/mL produces the most pronounced improvement in cartilage repair among four tested concentrations (1, 3, 10, and 30 mg/mL) (161). By contrast, long-term VC administration resulting in excessively high serum levels exacerbates spontaneous OA in guinea pigs, accompanied by increased active transforming growth factor-β (TGF-β) expression in marginal osteophytes and elevated synovial fluid COMP levels (162). These findings suggest that VC may have an optimal effective range in OA, beyond which excessive supplementation may be ineffective or potentially detrimental.

VC-based combination strategies further support its mechanistic relevance to OA. Intra-articular co-administration of VC and Mg^2+^ additively alleviates joint degradation and pain in a mouse OA model, with sustained effects after treatment cessation. Mechanistically, VC enhances Mg^2+^-mediated activation of HIF-1α, promotes M2 polarization of synovial macrophages, and inhibits osteophyte formation and pain-related neuropeptide production (163). *In vitro*, VC combined with β-caryophyllene and D-glucosamine acts synergistically to suppress macrophage-mediated inflammatory responses in primary human chondrocytes and promote COL2A1 and ACAN expression (164). VC also enhances the anticatabolic effects of hyaluronic acid in IL-1β-stimulated chondrocytes, leading to reduced MMP3 and MMP9 expression (165). However, an intra-articular antioxidant formulation containing VC, quercetin, and deferoxamine does not retard structural disease progression in an advanced post-traumatic OA rat model, suggesting that disease stage may influence therapeutic efficacy (166). In addition, VC has been incorporated into several cell-based cartilage repair strategies. It promotes the long-term expansion and chondrogenic potential of BMSCs while reducing cellular senescence and heterogeneity (167). In a minipig model of full-thickness cartilage defects, transplantation of BMSCs pretreated with VC and ferumoxytol enhances chondrogenesis, inhibits apoptosis, and promotes hyaline-like cartilage regeneration (168), with greater efficacy than iron-labeled BMSC implants without VC pretreatment (169). VC also facilitates the formation of chondrogenic cell sheets from adipose-derived stem cells, which survive long term in the synovium and delay cartilage degeneration after intra-articular injection in rabbit OA models (170). Similarly, VC-treated scaffold-free costal chondrocyte constructs enhance cartilage matrix deposition and chondrogenic gene expression, thereby promoting native-like cartilage regeneration in rat osteochondral defects (171). These findings support further investigation of VC as a bioactive component in cartilage repair and regenerative strategies.

### 5.3 RA

RA is a chronic autoimmune disease characterized by synovial hyperplasia, joint inflammation, cartilage degradation, and bone destruction (172). Although evidence regarding the role of VC in RA remains limited, preclinical studies suggest that VC may modulate RA-related immune and inflammatory responses through multiple mechanisms. In a collagen-induced arthritis (CIA) mouse model, VC reduces autoantibody production and attenuates arthritis severity. Mechanistically, VC blocks B-cell cycle progression, promotes apoptosis, and suppresses differentiation into autoreactive plasma cells, an effect mediated at least partly through inhibition of signal transducer and activator of transcription 3 (STAT3) signaling pathway (173). RA pathogenesis is also associated with gut microbiota imbalance, which may promote VC degradation and aggravate disease activity (174). Conversely, VC supplementation restores gut microbial diversity in CIA mice, alleviates intestinal injury, and reduces joint swelling, cartilage and bone destruction, and inflammatory cell infiltration (175). These findings suggest that gut microbiota may represent an additional pathway linking VC to RA-related inflammation and point to the gut–musculoskeletal axis as a potential target for understanding VC-related musculoskeletal pathology. VC also regulates macrophage-mediated inflammation and pyroptosis in RA. In CIA rats, combined treatment with VC and nitrate reduces pro-inflammatory cytokine levels in serum and synovial tissue, alleviates bone destruction, and suppresses macrophage infiltration and pyroptosis (176). In lipopolysaccharide-stimulated THP-1 macrophages, VC inhibits NF-κB expression and activation in a dose-dependent manner, thereby decreasing TNF-α and IL-1β production. Compared with nitrate alone, the combined treatment further reduces NLR family pyrin domain-containing 3 (NLRP3), cleaved caspase-1, and N-GSDMD levels (176). These findings suggest that VC may attenuate macrophage inflammation and pyroptosis by inhibiting NF-κB signaling and indirectly suppressing NLRP3 inflammasome activation. Beyond its direct immunomodulatory effects, VC-based delivery systems provide additional preclinical support for RA-targeted interventions. VC-functionalized transethosomes loaded with sinomenine hydrochloride enhance transdermal permeability and enable targeted drug delivery to oxidative stress sites in RA joints, thereby attenuating RA-related inflammation with efficacy comparable to that of gastric sinomenine hydrochloride administration (177). Similarly, an L-ascorbate palmitate-based nanoparticle delivery platform for triptolide suppresses synovitis and bone erosion, alleviates paw swelling and deformity in CIA mice, and reduces the hepatic and renal toxicity associated with triptolide monotherapy (178). In addition, an injectable hydrogel incorporating VC and 9-aminoacridine promotes collagen production through TET2 upregulation and suppression of pro-inflammatory cytokine expression in CIA rats, thereby facilitating the repair of inflammation-induced cartilage and joint damage (179).

### 5.4 Sarcopenia

Sarcopenia is an age-related, progressive, and generalized skeletal muscle disorder characterized by accelerated loss of muscle mass, strength, and/or physical performance (180). It is associated with adverse health outcomes, including falls, fractures, functional decline, frailty, and mortality (181). Preclinical evidence suggests that VC plays an important role in maintaining skeletal muscle mass and function (182). In senescence marker protein-30 (SMP30)-knockout mice, which are unable to synthesize VC, VC deficiency exacerbates oxidative stress, reduces muscle mass and physical performance, and decreases myofiber cross-sectional area. These changes are accompanied by upregulation of muscle atrophy-related genes, including forkhead box O1 (FOXO1), atrogin-1/muscle atrophy F-box (MAFbx), and muscle RING finger 1 (MuRF1). VC re-supplementation completely reverses these adverse effects, supporting a protective role of VC against muscle atrophy in SMP30-knockout mice of both sexes (36, 183). In myotubes differentiated from human primary myoblasts, VC attenuates H_2_O_2_-induced atrophy by suppressing the FOXO1/MuRF1/MAFbx axis of the ubiquitin–proteasome system and reducing oxidative stress-induced autophagy activation (184, 185). In addition, aquaculture models suggest that VC supplementation may enhance immune responses and muscle regeneration under stress conditions while promoting collagen and elastin deposition in muscle, indicating a potential role in muscle repair and structural integrity through regulation of ECM-related structural protein metabolism (186, 187).

Mitochondrial quality control and functional integrity are central to skeletal muscle health and sarcopenia management (188, 189). In C2C12 myoblasts, VC restores mitochondrial membrane potential and suppresses mitochondrial dysfunction (190). VC also attenuates age-related mitochondrial functional decline by alleviating mitochondrial oxidative damage (191). In teleost fish during fasting-refeeding recovery, VC supplementation facilitates muscle recovery by enhancing mitochondrial biogenesis and energy metabolism, thereby promoting muscle growth and increasing myofiber diameter (192). Notably, VC enhances mitochondrial biogenesis-related gene expression in myogenic cells during proliferation and early differentiation, although this effect is not maintained at later differentiation stages (53). This stage-dependent response may be related to changes in SVCT2-mediated VC transport. During C2C12 myoblast differentiation into myotubes, plasma membrane SVCT2-mediated VC transport remains stable, whereas mitochondrial SVCT2 function becomes impaired. Mitochondrial SVCT2 is highly expressed during the proliferative phase, when metabolic and antioxidant demands are elevated, but progressively declines during differentiation and becomes undetectable in late-stage myotubes. Consequently, elevated VC concentrations or prolonged exposure remain insufficient to effectively clear mitochondrial superoxide in myotubes, indicating severely impaired mitochondrial VC uptake capacity (193). These findings suggest that VC supplementation strategies for muscle health and sarcopenia should consider not only systemic VC status but also mitochondrial function and SVCT2-dependent tissue uptake capacity.

## 6 Clinical evidence for the role of VC in musculoskeletal disorders

### 6.1 OP and bone health

Observational studies have reported positive associations between VC status and bone health. Cross-sectional studies show that higher dietary VC intake, adequate plasma VC levels, and long-term VC supplement use are associated with higher BMD in postmenopausal women (194–196). Increased VC intake has also been linked to reduced bone loss over 4 years in elderly men (197). In a 17-year follow-up study, greater total and supplemental VC intake is associated with a lower risk of hip and non-vertebral osteoporotic fractures in older adults (198). Meta-analyses further suggest that greater consumption of VC-rich foods is inversely associated with the risk of hip fracture, OP, and BMD loss (199, 200). A dose-response meta-analysis indicates that each 50 mg/day increase in VC intake is associated with an approximately 5% lower risk of hip fracture (201). However, recent evidence from Mendelian randomization analyses does not support a clear causal relationship between circulating VC levels and OP risk in adults (202). Overall, observational evidence generally supports a favorable association between VC status and bone health, but causality remains uncertain. These associations may also be influenced by age, smoking status, baseline nutritional status, and overall dietary patterns 2studies remain limited and often involve multi-nutrient formulations. In a small randomized study of 44 osteopenic postmenopausal women, 12-month supplementation with an antioxidant formulation containing VC (30 mg) plus calcium and vitamin D modestly improves estimated BMD compared with calcium and vitamin D alone (204). A double-blind, placebo-controlled trial in 90 older adults reports that VC (1,000 mg/day) combined with vitamin E (400 IU/day) for 12 months attenuates lipid peroxidation associated with hip BMD loss (205). More recently, a randomized trial in 78 menopausal women shows that a multi-nutrient dairy product enriched with VC (100 mg per 150 g) maintains BMD and favorably modulates bone turnover markers (206). These findings suggest potential benefits of VC-containing multi-nutrient interventions for bone health, although the independent contribution of VC cannot be determined. Evidence from VC-only interventions is particularly scarce. A 13-week study in 75 patients with OP reports that VC supplementation (500 mg/day) reduces serum TRAP levels and improves oxidative stress markers (207). Another 12-week study in 68 elderly postmenopausal women with OP shows that VC supplementation (500 mg/day) does not significantly improve bone formation markers, although slight reductions in markers of bone resorption are observed (208). Notably, neither study uses a placebo-controlled VC-only design or directly assesses bone outcomes such as BMD or fracture risk. Overall, current clinical evidence remains insufficient to support a definitive independent protective effect of VC against OP.

### 6.2 OA, RA, and joint health

Although VC is generally considered beneficial for joint health, its clinical relationship with OA remains inconsistent. Cross-sectional studies report associations between VC intake and either lower or higher OA prevalence (209–211), while higher circulating VC levels have been associated with an increased risk of incident OA (212). By contrast, case-control studies show lower dietary VC intake and circulating VC levels in patients with OA than in healthy controls, alongside greater oxidative stress and weaker antioxidant defense (213, 214). These discrepancies may partly reflect heterogeneity in population characteristics, outcome definitions, and VC exposure assessment, including dietary intake, supplement use, and circulating VC levels, as well as reverse causation and residual confounding in cross-sectional and case-control studies. A non-linear or threshold-dependent response may also contribute, given the reduced benefits or adverse effects of excessive exposure observed in preclinical models (160–162). The relationship may further differ between OA onset and progression. A prospective cohort study associates high dietary VC intake (≥200 mg/day) with lower risks of OA progression, cartilage loss, and knee pain, but not incident OA (215). However, another study suggests that VC supplement use may help prevent OA without delaying established disease progression (216). Collectively, these findings indicate that associations between VC and OA may vary according to disease stage and the method used to assess VC exposure.

Interventional studies have mainly focused on knee OA. An early randomized, double-blind, placebo-controlled crossover trial in 34 men with degenerative knee joint disease shows that a glucosamine/chondroitin formulation containing manganese ascorbate (228 mg/day) for 16 weeks improves OA pain and symptom scores (217). In 120 patients with early OA, adding a VC-containing multi-nutrient formulation to conventional nutritional therapy reduces pain scores, improves quality of life, and decreases analgesic use at 6 and 12 months, but has no significant effect on knee joint function (218). More recently, a trial in 70 patients with stage I–III primary knee OA shows that 3–6 months of supplementation with a VC-containing multi-nutrient formulation is non-inferior to combined glucosamine/chondroitin therapy for several symptomatic and imaging outcomes (219). Notably, a 12-week double-blind randomized controlled trial in 60 patients with moderate-to-severe knee OA uses low-dose VC (approximately 59.4 mg/day) as an active control and shows that a polyphenol formulation containing the same dose of VC is superior to VC alone in improving symptoms and inflammatory and oxidative stress markers (220). Differences in disease stage, intervention composition, comparators, duration, and outcome measures limit comparability across studies. Moreover, the frequent use of multi-component formulations prevents attribution of the observed effects to VC alone. Overall, VC status may be associated with OA progression, while VC-containing interventions may improve symptoms. However, evidence regarding OA onset remains inconsistent, and the independent contribution of VC remains unclear.

Compared with OA, clinical evidence linking VC to RA is more limited, but suggests a possible association with lower inflammatory burden and disease activity. Observational studies show that higher dietary VC intake and circulating VC levels are associated with a lower prevalence of RA (221–223), whereas lower VC intake is associated with a more pro-inflammatory dietary pattern and higher disease activity (224). Patients with RA often show increased oxidative stress and reduced antioxidant defense. A randomized study reports that baseline plasma VC, glutathione, and total thiol levels are lower in patients with RA than in healthy controls, whereas malondialdehyde (MDA) levels are higher. After 12 weeks of combined supplementation with antioxidants containing VC, vitamin A, and vitamin E in addition to conventional therapy, antioxidant indices increase, while MDA levels and disease activity show a greater reduction (225). More direct evidence comes from a VC add-on supplementation study, in which patients with RA receiving standard therapy plus VC (1 g/day) for 8 weeks show significantly reduced ROS and reactive nitrogen species levels and improved disease activity (226). A meta-analysis of randomized clinical trials further shows that VC supplementation reduces circulating IL-6 levels, providing indirect clinical support for its anti-inflammatory potential in RA (227). However, a randomized, double-blind, placebo-controlled crossover trial in 20 patients with RA shows that 4-week supplementation with quercetin plus VC (approximately 399 mg/day) does not significantly improve inflammatory markers, including TNF-α, IL-1β, IL-6, and C-reactive protein (CRP), or disease severity (228). These findings suggest that VC may have an adjunctive antioxidant and anti-inflammatory role in RA management. However, its clinical efficacy remains uncertain and may vary with dose, treatment duration, co-administered compounds, background therapy, and disease status.

### 6.3 Sarcopenia and muscle health

Nutritional intake plays an important role in the prevention and management of sarcopenia (229). Human studies show that skeletal muscle VC levels are closely related to dietary intake and plasma VC concentrations and are highly responsive to VC supplementation, suggesting that adequate VC status may help support skeletal muscle mass and function (29). Observational studies further show that higher dietary VC intake and circulating VC levels are associated with greater muscle mass, strength, and physical performance in middle-aged and older adults, as well as a lower risk of sarcopenia (230–233). Interventional evidence remains limited and largely involves combined nutritional formulations. A 12-week randomized, double-blind trial in 66 older adults with sarcopenia shows that fortified yogurt containing VC (500 mg/day), β-hydroxy-β-methylbutyrate, and vitamin D improves handgrip strength and gait speed, increases serum IGF-1, and reduces CRP and MDA levels (234). In patients with moderate-to-severe chronic obstructive pulmonary disease, 8-week consumption of a whey beverage containing VC (685 mg/day) and magnesium reduces IL-6 levels and improves muscle mass, handgrip strength, and quality of life (235). In patients with facioscapulohumeral muscular dystrophy, 17-week supplementation with a combined antioxidant formulation containing VC (500 mg/day), vitamin E, zinc, and selenium reduces oxidative stress and improves bilateral quadriceps strength and endurance (236). More VC-specific evidence comes from a randomized crossover study in patients with type 2 diabetes, in which oral VC supplementation (1,000 mg/day) for 16 weeks increases skeletal muscle VC concentration and SVCT2 protein expression and reduces skeletal muscle oxidative stress under hyperinsulinemic conditions (237). However, this study does not assess muscle mass or function. Overall, current evidence suggests that VC may be associated with muscle health and sarcopenia-related outcomes, potentially through reduced oxidative stress and inflammation. However, heterogeneous populations, varied outcomes, and frequent use of multi-nutrient formulations limit generalizability, and direct VC-only evidence in patients with sarcopenia remains lacking. **Table 3** summarizes human, animal, and mechanistic evidence on VC in major musculoskeletal disorders, with corresponding strength appraisals and clinical implications.

**Table 3:** Summary of evidence and clinical implications of VC in major musculoskeletal disorders.

Disorder	Human evidence	Animal evidence	Main mechanisms	Strength of evidence	Clinical implication
OP	Observational findings are generally favorable, but causality remains uncertain (194–202). Human interventions are limited, frequently use combined formulations, and do not establish an independent VC effect (204–208).	VC attenuates bone loss, promotes bone formation, and suppresses bone resorption across VC-deficient and osteoporotic animal models (35, 142, 143, 146, 149).	Promotion of osteoblastogenesis, suppression of osteoclastogenesis, and maintenance of bone cell homeostasis through antioxidant, epigenetic, and SVCT2-dependent mechanisms (35, 143–145, 148, 149).	Low to moderate	Adequate VC status may support bone health, but current evidence does not support VC-only treatment for OP.
OA	Associations with OA onset and progression are inconsistent (209–216). Combined interventions may improve symptoms or joint structure-related parameters, but the independent contribution of VC remains unclear (217–220).	VC mitigates cartilage degeneration and supports cartilage repair within an optimal dose range, whereas excessive exposure may be detrimental (160–163, 166, 168, 170, 171).	Attenuation of oxidative stress and inflammatory responses, inhibition of chondrocyte senescence and apoptosis, and maintenance of cartilage matrix homeostasis (156–160, 164, 165).	Low and inconsistent	Routine VC supplementation cannot currently be recommended because dose–response relationships, disease-stage-specific effects, and the independent contribution of VC remain unclear.
RA	Higher VC status is associated with lower inflammatory burden (221–224), but intervention studies are limited and mainly support an adjunctive role (225, 226).	VC or VC-based interventions alleviate arthritis progression and joint damage in CIA models (173, 175, 176, 178, 179).	Suppression of autoreactive B-cell responses, restoration of gut microbial homeostasis, and inhibition of NF-κB/NLRP3-mediated macrophage inflammation and pyroptosis (173, 176).	Low	VC may have adjunctive potential, but its independent clinical efficacy remains unproven.
Sarcopenia	Observational findings are generally favorable (230–233), but interventional evidence mainly comes from combined formulations, and VC-only studies in patients with sarcopenia are lacking (234–236).	VC deficiency induces muscle atrophy and functional impairment in SMP30-knockout mice, whereas VC supplementation reverses these effects (36, 183).	Attenuation of oxidative stress, ubiquitin–proteasome activation, and excessive autophagy, with preservation of mitochondrial function and promotion of mitochondrial biogenesis (53, 184, 185, 190).	Low to moderate	Maintaining adequate VC status may support muscle health, but current evidence does not support routine VC supplementation for sarcopenia.

Overall evidence strength was narratively appraised according to study design, consistency, directness to clinical outcomes, and translational relevance. Observational human evidence was generally considered low to moderate; small, uncontrolled, or multi-component human interventional studies were considered low; and animal and cell/mechanistic studies were treated as preliminary preclinical or mechanistic support. Overall ratings reflect the combined evidence for each disorder, with greater weight given to direct human interventional evidence.

### 6.4 Dose–response relationship and translational considerations

Determining the optimal dose of VC is one of the key unresolved issues in translating VC-related findings to musculoskeletal health. Current evidence suggests that the effects of VC do not follow a simple linear dose-response relationship, but may instead show a U-shaped or threshold-dependent pattern, in which both VC deficiency and excessive exposure may adversely affect musculoskeletal tissues. Current dietary recommendations mainly aim to achieve adequate VC status, corresponding to a plasma VC concentration of approximately 50 μmol/L, and a daily VC intake of approximately 200–400 mg can bring plasma levels close to saturation (203, 238). Across the clinical studies summarized in this review, VC exposure ranges from approximately 30 to 1,000 mg/day, with intervention durations ranging from 4 weeks to 12 months. Although most studies use doses above the recommended dietary intake, related adverse events are rarely reported, with limited safety reporting across studies. This may partly reflect the saturable absorption kinetics of oral VC, whereby plasma and tissue VC levels do not increase linearly with increasing oral doses (24).

VC bioavailability can be influenced by transporter expression, disease status, intervention duration, route of administration, and formulation. SVCT2-mediated tissue uptake may be particularly important. For example, in postmenopausal women, oral administration of 10 g VC per day for 4 weeks does not achieve the expected antioxidant effect (239). By contrast, oral VC supplementation (1,000 mg/day) for 16 weeks increases skeletal muscle VC levels and SVCT2 protein expression and reduces skeletal muscle oxidative stress in patients with type 2 diabetes (237), whereas the same dose for 6 weeks increases skeletal muscle SVCT2 expression without clearly altering redox markers in healthy men (240). These findings suggest that the response to VC supplementation may depend on disease context, intervention duration, and tissue uptake capacity. Oral VC mainly corresponds to nutritional supplementation or correction of deficiency, whereas intravenous administration can bypass saturable intestinal absorption and achieve pharmacological plasma concentrations far above those attainable with routine oral supplementation (21). Evidence for intravenous VC in musculoskeletal health remains very limited. A clinical study in patients undergoing total knee replacement reports that postoperative intravenous VC (15 g) reduces inflammatory markers, suggesting potential anti-inflammatory benefits during recovery, although its relevance to chronic musculoskeletal disorders remains uncertain (241). Emerging formulations may also alter VC bioavailability. For example, liposomal VC may enhance absorption through SVCT-independent mechanisms (242, 243) and shows potential for improving post-disease physical performance and muscle functional recovery in short-term interventions (244). Liquid VC formulations may also have higher absorption rates than tablets or capsules, with stronger absorption responses observed in individuals with greater muscle mass (245). In human OA chondrocyte studies, donor characteristics such as age and BMI may further influence responses to VC intervention (246). It should be noted that VC concentrations used in cell experiments and mg/kg doses used in animal studies are difficult to directly align with systemic or tissue exposure achieved through routine oral supplementation in humans. Therefore, their clinical relevance should be interpreted with caution. Overall, current evidence remains insufficient to define an optimal VC dose for musculoskeletal health, and nutritional supplementation, deficiency correction, and pharmacological administration should be considered distinct exposure contexts.

## 7 VC and exercise in musculoskeletal health

Exercise is an effective intervention for maintaining musculoskeletal health and managing musculoskeletal disorders (247, 248). For patients with musculoskeletal disorders, combining exercise with nutritional interventions has attracted increasing interest, particularly when pain, weakness, frailty, or functional impairment limits exercise volume or intensity. As a common component of nutritional supplements, VC may be a candidate for modulating exercise-induced musculoskeletal adaptation. Preclinical evidence provides some support for this possibility. *In vitro*, appropriate mechanical stimulation appears to support VC-mediated cartilage ECM development and improvements in mechanical properties, whereas VC may attenuate static loading-induced chondrocyte degeneration (249, 250). In animal models, VC alone or combined with vitamin E reduces exercise-induced oxidative damage and improves muscle- or bone-related outcomes under oxidative stress conditions (251, 252). However, antioxidant supplementation during exercise may not always be beneficial, as ROS also act as adaptive signaling molecules (253). Indeed, animal studies suggest that high-dose VC supplementation before or during exercise may blunt exercise-induced mitochondrial biogenesis signaling and proteostatic adaptations despite reducing oxidative stress (254, 255).

Current clinical evidence on VC combined with exercise remains limited and has largely focused on exercise adaptation rather than musculoskeletal disease outcomes. In older adults, acute oral VC supplementation improves skeletal muscle perfusion and oxidative metabolic capacity during exercise, changes that may be important for enhancing physical performance (256, 257). In patients with chronic obstructive pulmonary disease, acute supplementation with a VC-containing antioxidant formulation increases plasma VC levels and reduces exercise-related free radical signals, particularly in individuals with higher baseline oxidative stress, but does not improve quadriceps endurance or post-exercise muscle fatigue (258). Long-term studies show mixed results. In older adults, adding VC (1,000 mg/day) and vitamin E (400 IU/day) to resistance training improves plasma antioxidant status and increases muscle mass, but confers no additional benefit over resistance training alone (259). Among postmenopausal women, either antioxidant supplementation with VC (1,000 mg/day) and vitamin E (600 mg/day) or resistance training alone prevents lumbar spine BMD loss, while combining them provides no additional benefit (260). Conversely, in older men undergoing 12 weeks of resistance training, the combination of VC (1,000 mg/day) and vitamin E (235 mg/day) attenuates gains in lean mass and rectus femoris muscle thickness (261) and diminishes improvements in BMD (262). These effects have been attributed, at least in part, to excessive antioxidant supplementation limiting ROS-mediated adaptive signaling. During high-intensity interval training, the same antioxidant regimen also attenuates ROS-mediated changes in inflammatory, calcium-related, and mitochondrial signaling pathways in skeletal muscle of older adults (263). Importantly, individuals with insufficient or deficient VC status may be more likely to benefit from targeted supplementation during exercise interventions (264, 265). Recent evidence further shows that, in older women with sarcopenia and inadequate VC levels, supplementation with VC (1,000 mg/day) and vitamin E (335 mg/day) during 12 weeks of resistance training increases muscle mass and strength, improves systemic redox status, and reduces pro-inflammatory cytokine levels compared with placebo (266). These findings suggest that baseline VC status may be an important determinant of whether antioxidant supplementation supports or blunts exercise-induced musculoskeletal adaptation.

Supplementation timing may also influence whether VC supports or blunts ROS-mediated exercise adaptation. Administering VC after the peak of exercise-induced ROS signaling, guided by oxidative damage markers such as MDA and protein carbonyls, may help optimize supplementation timing (267). One study suggests that VC should be administered at least 1 h after exercise cessation, although the optimal timing may vary according to exercise intensity and individual characteristics (268). The exercise prescription itself may also be relevant. Resistance training is more effective for maintaining muscle mass and bone density, whereas lower-intensity endurance training may be more suitable for patients with OA and RA (269). Different exercise modalities induce distinct metabolic and oxidative stress responses (270, 271), and multimodal exercise programs may therefore influence the effects of VC supplementation. In older women undergoing combined core stability and aerobic training, short-term VC supplementation (1,000 mg/day for 6 weeks) improves exercise-induced inflammatory adaptation (272). In addition, the redox-sensitive transporter SVCT2 is regulated by contraction-mimetic signals (273–275), suggesting a potential role in exercise-related VC responses, although this variable has rarely been incorporated into existing studies. Together, these findings indicate that the effects of VC on exercise adaptation may vary with baseline VC status, supplementation dose and timing, tissue uptake capacity, and exercise modality. Representative clinical studies investigating VC–exercise interactions in musculoskeletal health are summarized in **Table 4**.

**Table 4:** Representative clinical studies on VC–exercise interactions in musculoskeletal health.

Study population	Age	Baseline VC level (μmol/L)	Nutritional supplementation	Exercise prescription	Weeks of treatment	Outcomes	Ref
Sedentary older adults	65.6 ± 3.8 years old	20.8 ± 14.4	VC (1,000 mg/d) and vitamin E (400 IU/d)	Resistance training: supervised high-intensity program, 3 sessions/week	24 weeks	Improved plasma antioxidant status and muscle mass, but provided no additional benefit over exercise alone.	(259)
Postmenopausal women	66.1 ± 3.3 years old	/	VC (1,000 mg/d) and vitamin E (600 mg/d)	Resistance training: 7 exercises, 3 sets of 8 repetitions at 80% of 1 RM, 3 sessions/week	24 weeks	Prevented BMD loss, but combined supplementation and exercise provided no additional benefit.	(260)
Sedentary older men	68 ± 6 years old	63.3 ± 18.1	VC (1,000 mg/d) and vitamin E (235 mg/d)	Resistance training: 8–10 RM performed twice per week, alternating with 3–5 RM or 13–15 RM performed once per week, 3 sessions/week	12 weeks	Attenuated resistance exercise-induced increases in BMD and muscle mass.	(261, 262)
Older men	65 years old	/	VC (1,000 mg/d) and vitamin E (235 mg/d)	High-intensity interval exercise: 4–6 bouts of 30-s all-out cycling with 4-min rest intervals, 3 sessions/week	3 weeks	Blunted ROS-induced signaling of exercise adaptation in skeletal muscle.	(263)
Older women with sarcopenia	66.1 ± 4.1 years old	32.5 ± 10.6	VC (1,000 mg/d) and vitamin E (335 mg/d)	Resistance training: 3 sessions/week; 2 moderate sessions (OMNI-RES: 6–7; 8–10 repetitions), 1 session varied between light (OMNI-RES: 4–5) and heavy (OMNI-RES: 8–9) every second week	12 weeks	Enhanced muscle mass and strength while reducing oxidative damage and inflammatory markers.	(266)
Older women	72.8 ± 5.3 years old	/	VC (1,000 mg/d)	Multimodal exercise program (core stability and aerobic training), 60 min/session, 3 sessions/week	6 weeks	Positively influenced exercise-induced inflammatory adaptation.	(272)
Sedentary older adults	65.5 ± 3.7 years old	19.2 ± 13.7	VC (1,000 mg/d) and vitamin E (600 mg/d)	Resistance training: 7 exercises, 3 sets of 8 repetitions at 80% of 1 RM, 3 sessions/week	24 weeks	Significantly increased muscle mass only in the combined group.	(276)

RM, repetition maximum; OMNI-RES, OMNI-Resistance Exercise Scale.

## 8 Research gaps and future directions

Despite progress in understanding the role of VC in musculoskeletal health, current evidence remains predominantly preclinical, while direct clinical evidence is limited. Responses to VC may vary with baseline VC status; dose, formulation, route of administration, and intervention duration; tissue uptake capacity; and individual characteristics such as age, sex, nutritional status, and comorbidities. Disease stage and inflammatory or oxidative stress burden may further modify responsiveness. In exercise settings, supplementation timing and exercise modality may also determine whether VC supports or blunts adaptation. However, few adequately powered clinical trials have systematically considered these factors, and most interventions use multi-nutrient formulations, leaving the independent effects and clinical relevance of VC uncertain. Differences in VC exposure assessment and outcome definitions further limit comparability across studies.

Addressing these gaps will require well-designed, adequately powered, placebo-controlled randomized trials of VC-only supplementation. Participants should be stratified by baseline VC status, disease stage, demographic characteristics, comorbidities, and inflammatory or oxidative stress burden to identify populations most likely to benefit. To define dose–response relationships, trials should systematically compare dose, formulation, route, timing, and duration while measuring achieved circulating or tissue VC concentrations, transporter-related indicators such as SVCT2 expression, and safety outcomes. Studies combining VC with exercise should use factorial designs comparing VC alone, exercise alone, and their combination to quantify additive, synergistic, or attenuating interactions. Concurrent monitoring of exercise-induced oxidative damage markers may help optimize supplementation dose and timing, while exercise prescriptions should systematically evaluate frequency, intensity, duration, and modality according to disease-specific characteristics. Finally, standardized VC exposure assessments and clinically meaningful endpoints, including BMD, fracture risk, joint structure, pain, muscle mass and function, physical performance, and musculoskeletal tissue healing, should be prioritized over surrogate biomarkers alone. These efforts will be essential to define the clinical relevance of VC and determine its potential role in maintaining musculoskeletal health and preventing or managing musculoskeletal disorders.

## 9 Conclusions

VC contributes to musculoskeletal homeostasis through antioxidant defense, anti-inflammatory activity, collagen synthesis, epigenetic regulation, modulation of cell proliferation and differentiation, and maintenance of mitochondrial function and energy metabolism. These actions support the development, maintenance, and repair of muscle, bone, cartilage, and tendon and influence pathological processes underlying musculoskeletal disorders. VC also shows promise as a bioactive component of cell-based and tissue-engineering strategies for musculoskeletal repair and regeneration. However, current evidence remains predominantly mechanistic and preclinical, whereas human interventional studies are limited, heterogeneous, and frequently involve multi-nutrient formulations. Taken together, available evidence supports a biologically important role for VC in musculoskeletal health and provides a strong rationale for evaluating its preventive, therapeutic, and regenerative potential. Further translational validation is therefore required before VC can be incorporated into evidence-based musculoskeletal care.

## Glossary

OP: Osteoporosis
OA: Osteoarthritis
RA: Rheumatoid arthritis
VC: Vitamin C
GULO: L-gulonolactone oxidase
DHA: Dehydroascorbic acid
SVCTs: Sodium-dependent vitamin C transporters
MuSCs: Muscle stem cells
BMSCs: Bone marrow mesenchymal stem cells
MRFs: Myogenic regulatory factors
MyoD: Myoblast determination protein 1
Myf5: Myogenic factor 5
MyoG: Myogenin
CSRP3: Cysteine and glycine-rich protein 3
ERK: Extracellular signal-regulated kinase
IGF-1: Insulin-like growth factor-I
KDM7A: Lysine demethylase 7A
TET: Ten-eleven translocation
mTOR: Mechanistic target of rapamycin
PI3K: Phosphoinositide 3-kinase
Akt: Protein kinase B
p70S6K: p70 ribosomal S6 kinase
Pax7: Paired box protein 7
ECM: Extracellular matrix
ALP: Alkaline phosphatase
BMP-2: Bone morphogenetic protein-2
COL-I: Type I collagen
OCN: Osteocalcin
BMD: Bone mineral density
Osx: Osterix
PHD: Prolyl hydroxylase domain
EB1: End-binding protein 1
Runx2: Runt-related transcription factor 2
BSP: Bone sialoprotein
MMPs: Matrix metalloproteinases
RANKL: Receptor activator of nuclear factor κB ligand
NF-κB: Nuclear factor kappa B
TRAP: Tartrate-resistant acid phosphatase
SOX9: SRY-box transcription factor 9
ACAN: Aggrecan
COMP: Cartilage oligomeric matrix protein
HIF-1α: Hypoxia-inducible factor 1α
ROS: Reactive oxygen species
OPN: Osteopontin
TNF-α: Tumor necrosis factor-α
CaMKIIα: Calcium/calmodulin-dependent serine/threonine kinase IIα
CREB: cAMP-response element-binding protein
ATF4: Activating transcription factor 4
MAPK: Mitogen-activated protein kinase
TGF-β: Transforming growth factor-β
STAT3: Signal transducer and activator of transcription 3
NLRP3: NLR family pyrin domain-containing 3
FOXO1: Forkhead box O1
MAFbx: Atrogin-1/muscle atrophy F-box
MuRF1: Muscle RING finger 1
MDA: Malondialdehyde
CRP: C-reactive protein
