# Supplementary material for: Insights into vitamin C in musculoskeletal physiology and disorders: mechanisms and translational perspectives
Source: Front Immunol. 2026 Aug 17;17:1899148. doi: 10.3389/fimmu.2026.1899148 (PMC13527207; doi:10.3389/fimmu.2026.1899148)
Supplement: Supplementary file 1 [file DataSheet1.pdf]

Supplementary Table S1. Preclinical evidence for VC in musculoskeletal disorders.

Study type	Dosage and administration of VC	Target cell/tissue	Mechanism	Biological functions	Ref
**OP**					
In vivo	VC-supplemented drinking water, 1000 mg/L	OVX-induced OP model in SD rats	• ALP, OPN, BMD↑<br>• Antioxidant capacity↑<br>• TRAP, DPD↓	Reversed bone loss in osteoporotic rats.	(142)
In vivo<br>In vitro	VC-supplemented drinking water, 5 mg/day	OVX-induced OP model in C56/BL6 mice<br>BMSCs from VC-treated mice	• BV/TV, BMD, Tb.N↑<br>• MS, MAR, BFR↑<br>• OCN, BSP, BMP-2, Runx2, Osx, ALP↑	Prevented post-ovariectomy bone loss.	(143)
In vitro	VC or DHA, 10, 20, or 50 μg/mL	Osteoclast precursors treated with M-CSF and RANKL	• RANK, c-Fos, c-Jun, p-ERK1/2↓	Inhibited osteoclast formation.	(144)
In vivo	Oral gavage of VC, 3, 7.5, or 15 mg/kg/day	OVX-induced OP model in Wistar rats	• BMP-2, SMAD 1/5/8, Runx2, OCN, COL-I, OPG↑<br>• RANK, RANKL, TRAP, cathepsin K↓<br>• Wnt3a, β-catenin, ATF4, p-AKT, p-ERK, p-p38, p-JNK↑	Prevented bone loss and increased calcium content and BMD in the tibia.	(146)
In vivo<br>In vitro	Oral gavage of magnesium ascorbyl phosphate, 20 mg/kg<br>Magnesium ascorbyl phosphate, 100 μM	OVX-induced OP model in C57BL/6J mice and femur defect model in SD rats<br>Skeletal stem and progenitor cells	• Runx2, Osx, COL-I, OPN, OCN, ALP, PCNA↑<br>• p-CaMKIIα, p-ERK1/2, p-CREB, c-Fos↑	Promoted bone formation via the CaMKII signaling pathway.	(35)
In vitro	VC, 250 μM	Sin-1-treated BMSCs	• Oxidative stress↓<br>• Autophagy↑	Protected BMSCs from oxidative damage by modulating autophagy.	(147,148)

			• Apoptosis↓		
In vivo	VC-supplemented drinking water, 3.3g/L	C57BL/6 mice	• Osteogenic differentiation↑<br>• Bone repair and regeneration↑	Preserved BMSC function and prevented osteoporosis.	(149)
In vitro	L-ascorbic acid 2-phosphate, 250 μM	BMSCs	• P16, P21↓		
**OA**					
In vitro	L-ascorbic acid 2-phosphate, 100 μmol/L	ROS-treated human chondrocytes and articular cartilage from patients with OA	• Telomere length, replicative capacity, GAG↑<br>• Oxidative damage↓	Inhibited oxidative stress-induced cartilage degeneration.	(156)
In vitro	VC, 100 or 200 μM	H_2O_2-treated C28/I2 human chondrocytes	• COL1A1, COL2A1, ACAN↑<br>• Nrf2, NF-κB, AP-1, MMP3↓<br>• Apoptosis↓	Inhibited oxidative stress-induced chondrocyte damage.	(157)
In vitro	VC, 100 or 200 μM	ATDC5 cells	• Mitochondrial superoxide, p16↓	Reversed chondrocyte senescence.	(159)
In vivo	Oral gavage of water-dissolved VC, 100, 200, or 300 mg/kg/day	MIA-induced OA model in Wistar rats	• Proteoglycan↑<br>• Bax, Cytochrome c, caspase-3/9↓	Protected chondrocytes against MIA-induced OA through multiple pathways.	(160)
In vitro	VC, 100 μM	SW1353 chondrosarcoma cells treated with 5 μM MIA	• IL-6, IL-17A, TNF-α, MMP1/3/13↓		
In vivo	Intra-articular injection of VC, 1, 3, 10, or 30 mg/mL	Cartilage defect model in New Zealand White rabbits	• COL-II, GAG↑<br>• TGF-β1, AKT, Nrf2, VEGF↓	Enhanced cartilage repair.	(161)
In vivo	Intra-articular injection of VC, 3 mg/mL, and/or $MgCl_2$, 0.5 mol/L	OA model induced by anterior cruciate ligament transection combined with partial medial meniscectomy in male C57BL/6 mice	• COL2A1, HIF-1α, SOX9↑<br>• MMP13, IL-6, COL10A1, TNF-α, PGE2↓	Synergistically alleviated joint pathology and pain in OA.	(163)
In vitro	VC, 2, 10, or 50 μM, combined with β-caryophyllene, 1 μM, and D-glucosamine, 10 or 20 μM	Primary human chondrocytes	• ROS↓<br>• IL-1β, NF-κB1, MMP-13↓<br>• COL-II, ACAN↑	Exerted anti-inflammatory, antioxidative, and chondroprotective effects.	(164)

In vitro	VC, 50 μg/mL, and/or hyaluronic acid, 100 μg/mL	IL-1β-stimulated chondrocytes	• MMP3/9, TNF-α, IL-1β↓	Synergistically enhanced the anticatabolic effect of hyaluronic acid.	(165)
In vivo	Transplantation of BMSCs pretreated with VC (0.2 mM) and ferumoxytol (100 μg Fe/mL)	Full-thickness cartilage defect model in minipigs	• COL-II↑<br>• Cartilage regeneration↑<br>• Apoptosis↓	Improved BMSC-mediated cartilage regeneration.	(168)
In vivo	Intra-articular injection of VC (50 mM)-assisted chondrogenic cell sheets	OA model in rabbits	• COL-II, SOX9, ACAN↑<br>• VEGF, MMP1/3, ADAMTS4↓	Inhibited OA progression.	(170)
In vivo	Implantation of VC (0.2 mM)-treated tissue-engineered constructs based on costal chondrocytes	Osteochondral defect model in SD rats	• SOX9, COL2A1, ACAN↑<br>• ICRS and O'Driscoll scores↑	Enhanced articular cartilage regeneration.	(171)
**RA**					
In vivo	Intraperitoneal injection of VC, 5 mg every other day	CIA model in C57BL/6 mice	• BV/TV, Tb.Th, Tb.N↑<br>• Tb.Sp, osteoclastogenesis↓	Inhibited RA by reducing autoantibody production.	(173)
In vitro	VC, 0, 20, 50, or 100 μM	LPS-stimulated B cells	• Autoantibody, antibody affinity maturation↓<br>• p-STAT3↓		
In vivo	Oral administration of VC, 100 mg/kg	CIA model in DBA/1 mice	• Gut microbial diversity and balance↑<br>• IL-6, TNF-α↓	Alleviated RA by modulating gut microbiota homeostasis.	(175)
In vivo	Drinking water supplemented with VC, 1 g/L, and/or sodium nitrate, 10 mmol/L	CIA model in Wistar rats	• BMD, BV/TV, Tb.Th, Tb.N↑<br>• Tb. Sp, BS/BV↓<br>• NF-κB, TNF-α, IL-1β, NLRP3, caspase-1, cleaved-caspase-1, GSDMD, N-GSDMD↓	Attenuated macrophage inflammation and pyroptosis.	(176)
In vitro	VC, 4 mM, and nitrate, 10 μM	LPS-stimulated THP-1 macrophages			

			• IL-10↑		
In vivo	VC-functionalized transethosomes loaded with sinomenine hydrochloride	RA model in New Zealand White rabbits and SD rats	• TNF-α, IL-6, ROS↓	Enhanced transdermal permeability and attenuated RA-related inflammation.	(177)
In vivo	L-ascorbate palmitate (50 mg/kg) and triptolide co-loaded nanoparticle	CIA model in Kunming mice	• GSH-Px↑<br>• IL-1β, IL-6, TNF-α, MDA↓<br>• ALT, AST, BUN, CRE↓	Alleviated RA and reduced triptolide-induced systemic toxicity.	(178)
In vivo	VC/9-aminoacridine co-loaded injectable hydrogel	CIA model in Wistar rats	• TET2↑<br>• IL-1β, NF-κB↓	Alleviated experimental inflammatory arthritis.	(179)
**Sarcopenia**					
In vivo	VC-supplemented drinking water, 1.5 g/L	SMP30-knockout mice	• ROS, FOXO1, MAFbx, MuRF1↓<br>• Muscle mass and physical performance↑	VC deficiency induced muscle atrophy and impaired physical capacity; both were reversed by VC supplementation.	(36,183)
In vitro	VC, 0.14 mM	H2O2-treated myotubes from patients with COPD	• ROS, PCO↓<br>• FOXO1, MAFbx, MuRF1↓	Reversed myotube atrophy.	(184)
In vitro	VC, 0.14 mM	Myoblasts and myotubes from patients with COPD	• ROS, PCO↓<br>• LC3 II/I, SQSTM1, BNIP3↓	Inhibited oxidative stress-induced autophagy activation.	(185)
In vitro	VC, 200 µM	C2C12 mouse myoblast cell line	• Mitochondrial membrane potential↑	Alleviated mitochondrial dysfunction.	(190)
In vivo	VC, 100, 200 or 400 mg/kg	Fasting and refeeding model in pacu (*Piaractus mesopotamicus*)	• PGC-1α, SDHA↑	Promoted muscle recovery during refeeding.	(192)
In vitro	VC, 300 µM	Primary mouse myogenic cells	• PGC-1α, TFAM, TFB1M, TFB2M↑	Promoted mitochondrial biogenesis during early myogenesis.	(53)

Abbreviations: ACAN, aggrecan; ADAMTS4, a disintegrin and metalloproteinase with thrombospondin motifs 4; AKT, protein kinase B; ALP, alkaline phosphatase; ALT, alanine aminotransferase; AP-1, activator protein 1; AST, aspartate aminotransferase; ATF4, activating transcription factor 4; Bax, BCL2-associated X protein; BFR, bone formation rate; BMD, bone mineral density; BMP-2, bone morphogenetic protein 2; BMSCs, bone marrow mesenchymal stem cells; BNIP3, BCL2-interacting protein 3; BSP, bone sialoprotein; BS/BV, bone surface/bone

volume; BUN, blood urea nitrogen; BV/TV, bone volume/tissue volume; CIA, collagen-induced arthritis; COL-I, type I collagen; COL-II, type II collagen; COPD, chronic obstructive pulmonary disease; CRE, creatinine; CREB, cAMP response element-binding protein; DHA, dehydroascorbic acid; ECM, extracellular matrix; ERK, extracellular signal-regulated kinase; FOXO1, forkhead box O1; FSHD, facioscapulohumeral muscular dystrophy; GAG, glycosaminoglycan; GSDMD, gasdermin D; GSH-Px, glutathione peroxidase; HIF-1α, hypoxia-inducible factor 1 alpha; IL, interleukin; JNK, c-Jun N-terminal kinase; KO, knockout; LC3, microtubule-associated protein 1 light chain 3; LPS, lipopolysaccharide; MAFbx, muscle atrophy F-box; MAR, mineral apposition rate; M-CSF, macrophage colony-stimulating factor; MDA, malondialdehyde; MIA, monosodium iodoacetate; MMP, matrix metalloproteinase; MS, mineralizing surface; MuRF1, muscle RING-finger protein 1; NF-κB, nuclear factor kappa-light-chain-enhancer of activated B cells; NLRP3, NOD-like receptor family pyrin domain-containing 3; Nrf2, nuclear factor erythroid 2-related factor 2; OA, osteoarthritis; OCN, osteocalcin; OP, osteoporosis; OPG, osteoprotegerin; OPN, osteopontin; Osx, osterix; OVX, ovariectomy/ovariectomized; PCO, protein carbonyl; PCNA, proliferating cell nuclear antigen; PGC-1α, peroxisome proliferator-activated receptor gamma coactivator 1 alpha; PGE2, prostaglandin E2; RA, rheumatoid arthritis; RANK, receptor activator of nuclear factor κB; RANKL, receptor activator of nuclear factor κB ligand; ROS, reactive oxygen species; Runx2, runt-related transcription factor 2; SD, Sprague–Dawley; SDHA, succinate dehydrogenase complex flavoprotein subunit A; Sin-1, 3-morpholinosydnonimine; SMAD, suppressor of mothers against decapentaplegic; SMP30, senescence marker protein 30; SOX9, SRY-box transcription factor 9; SQSTM1, sequestosome 1; SSPCs, skeletal stem and progenitor cells; STAT3, signal transducer and activator of transcription 3; Tb.N, trabecular number; Tb.Sp, trabecular separation; Tb.Th, trabecular thickness; TET2, ten-eleven translocation 2; TFAM, mitochondrial transcription factor A; TFB1M, mitochondrial transcription factor B1; TFB2M, mitochondrial transcription factor B2; TGF-β1, transforming growth factor beta 1; THP-1, human monocytic THP-1 cell line; TNF-α, tumor necrosis factor alpha; TRAP, tartrate-resistant acid phosphatase; VC, vitamin C; VEGF, vascular endothelial growth factor.
